# Supplementary material for: PRDM14 Drives OCT3/4 Recruitment via Active Demethylation in the Transition from Primed to Naive Pluripotency
Source: Stem Cell Reports. 2016 Nov 17;7(6):1072–86. doi: 10.1016/j.stemcr.2016.10.007 (PMC5161533; doi:10.1016/j.stemcr.2016.10.007)
Supplement: Document S1. Supplemental Experimental Procedures and Figures S1–S5 [file mmc1.pdf]

Stem Cell Reports, Volume 7

Supplemental Information

PRDM14 Drives OCT3/4 Recruitment via Active Demethylation in the Transition from Primed to Naive Pluripotency

Naoki Okashita, Yoshiaki Suwa, Osamu Nishimura, Nao Sakashita, Mitsutaka Kadota, Go Nagamatsu, Masanori Kawaguchi, Hiroki Kashida, Ayaka Nakajima, Makoto Tachibana, and Yoshiyuki Seki

Supplementary Figure S1

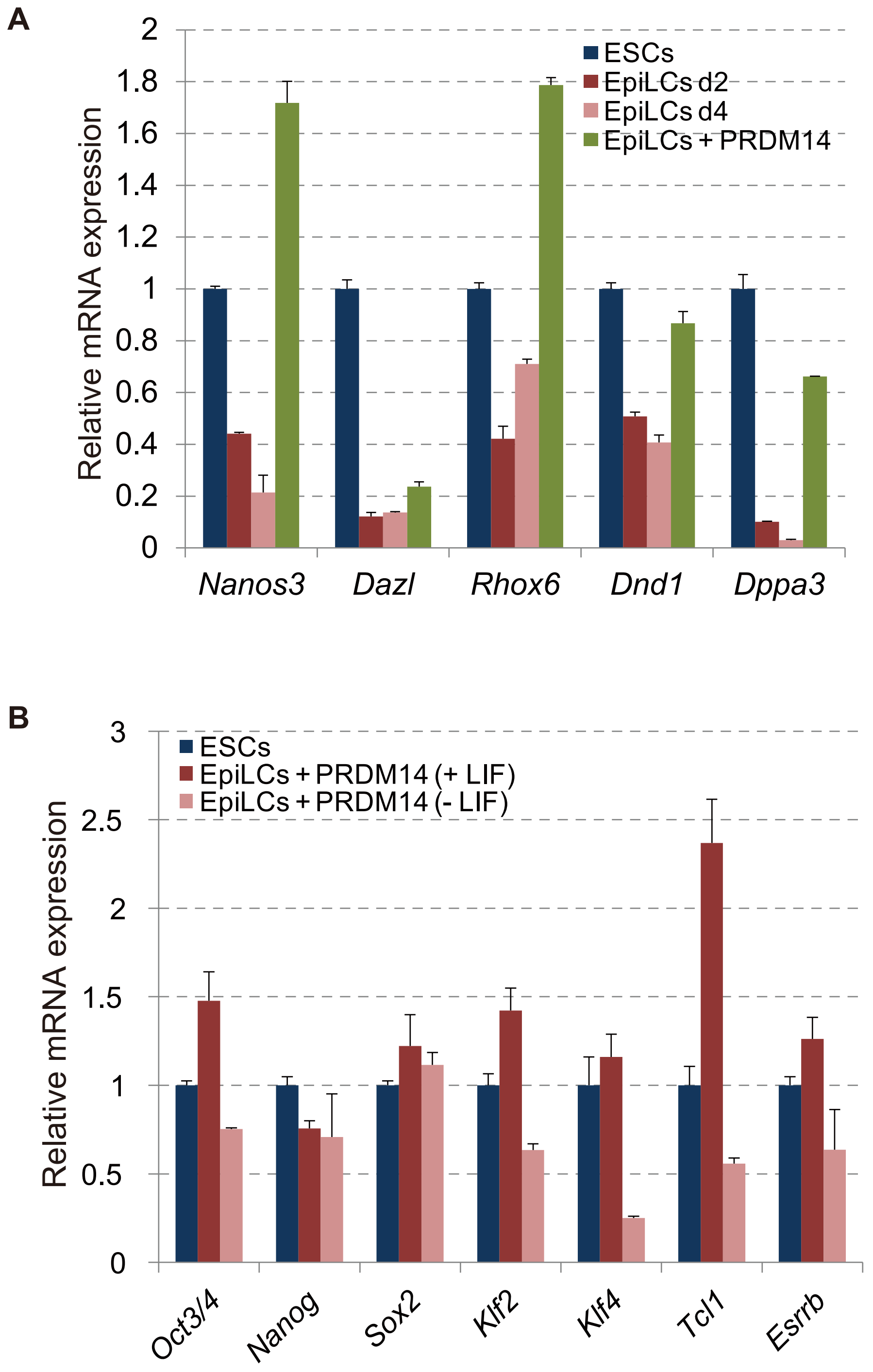

Supplementary Fig. 1 PRDM14 overexpression converts EpiLCs to ESC-like cells

(**A**) qRT-PCR anaysis of ealry PGC markers in ESCs, EpiLCs (d2), EpiLCs (d4) and EpiLCs + PRDM14. Error bar indicates + s.d. of technical duplicate. (**B**) qRT-PCR analysis of pluripotent-associated genes (*Oct3/4*, *Nanog*, *Sox2*, *Klf2*, *Klf4*, *Tcl1* and *Esrrb*) in ESCs and ESC-like cells converted from EpiLCs by PRDM14 in the presence or absence of LIF. Error bar indicates + s.d. of technical duplicate.

Supplementary Figure S2

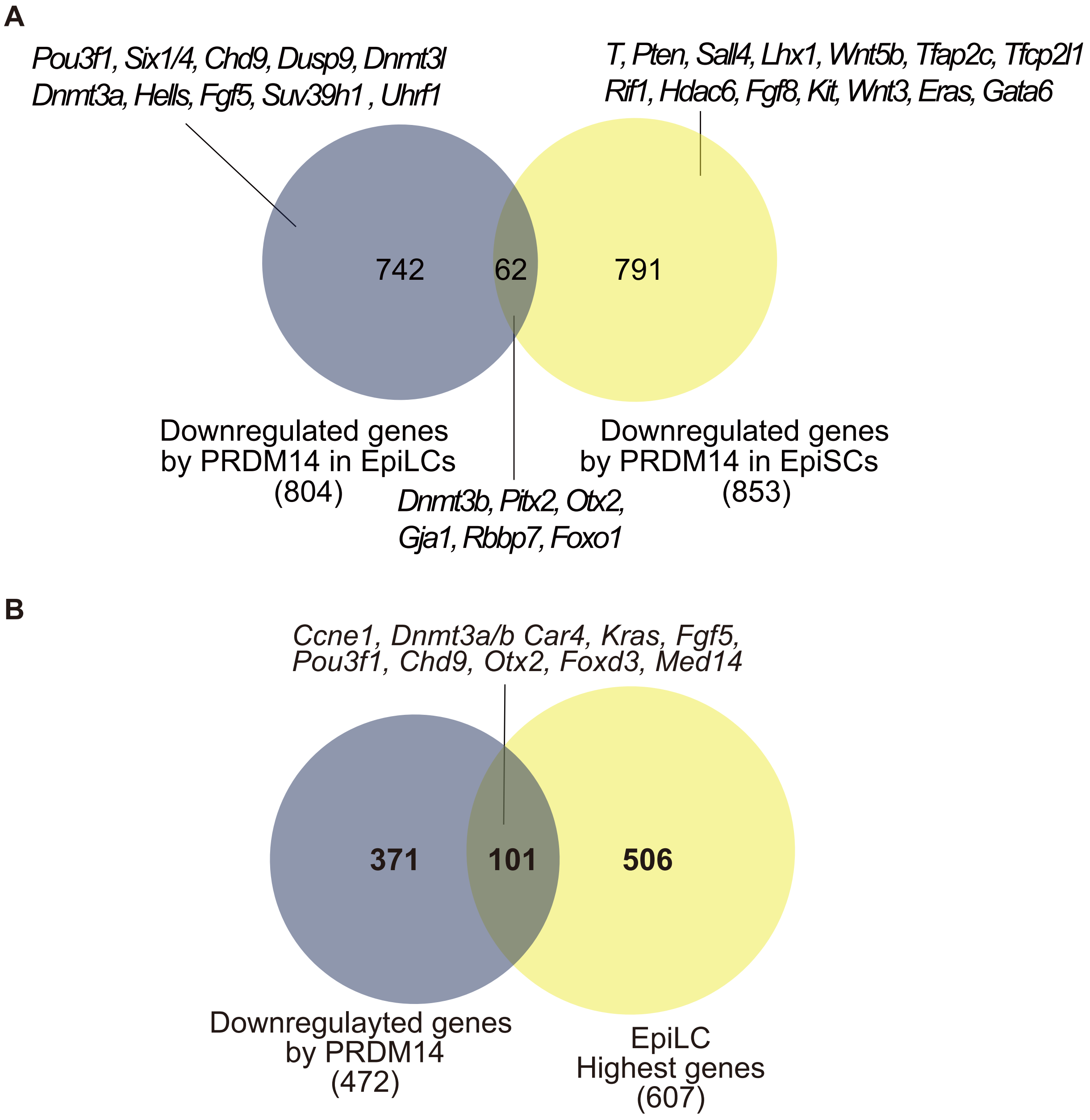

Supplementary Fig. 2 Venn diagram of downregulated genes by PRDM14

Supplementary Figure S3

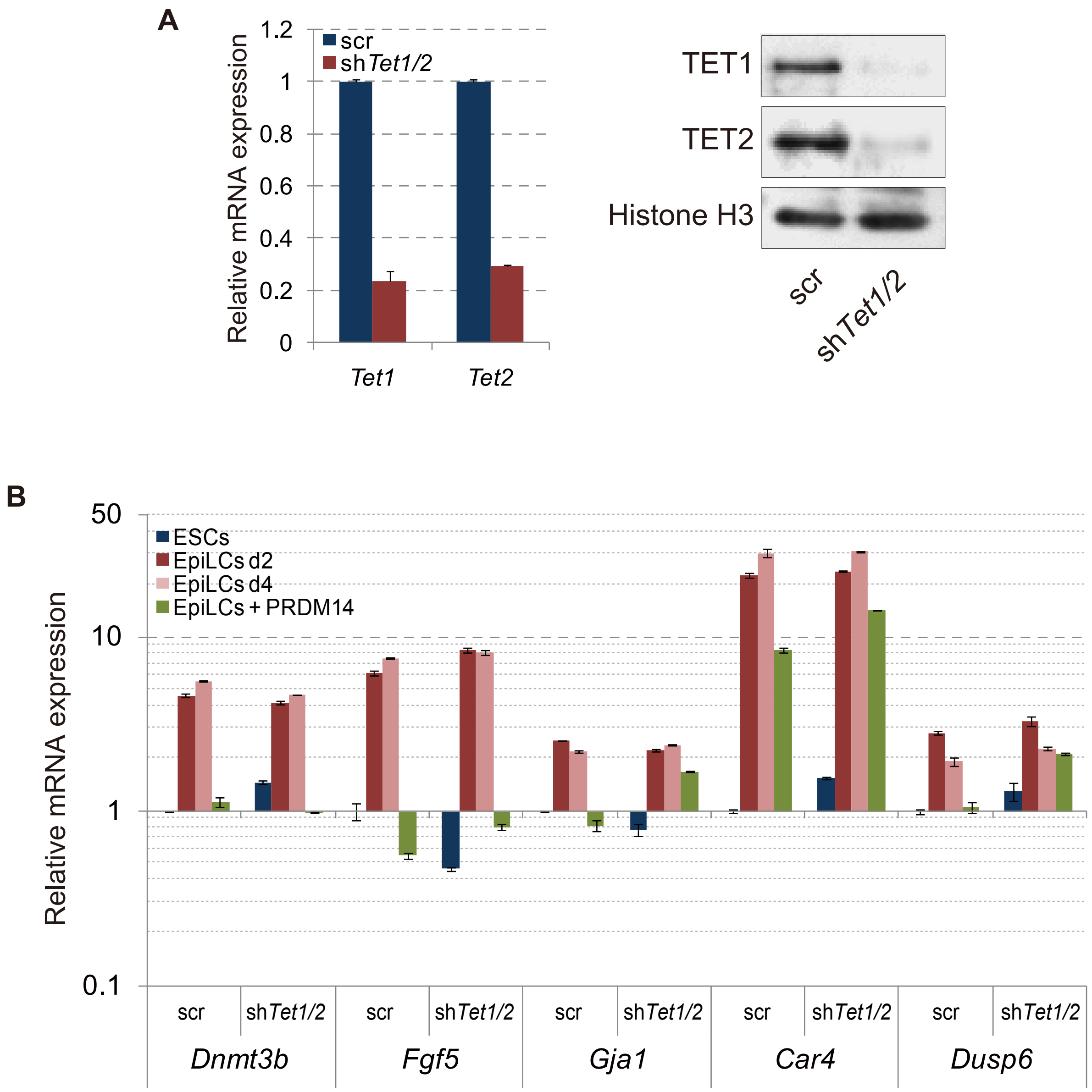

Supplementary Fig. 3 The varidation of knock down efficiency of *Tet1*, *Tet2* and qRT-PCR analysis

(**A**) qRT-PCR and Western blotting analysis of TET1 and TET2 expression in control (scr) and *Tet1/2* DKD (sh*Tet1/2*) Prdm14-inducible ESCs. (**B**) RT-PCR analysis of differentiation-associated genes and germline-specific genes expression in ESCs, EpiLCs d2, EpiLC d4 and EpiLCs + PRDM14 of control and *Tet1/Tet2* DKD cells. Error bar indicates ± s.d. of technical duplicate.

Supplementary Figure S4

A

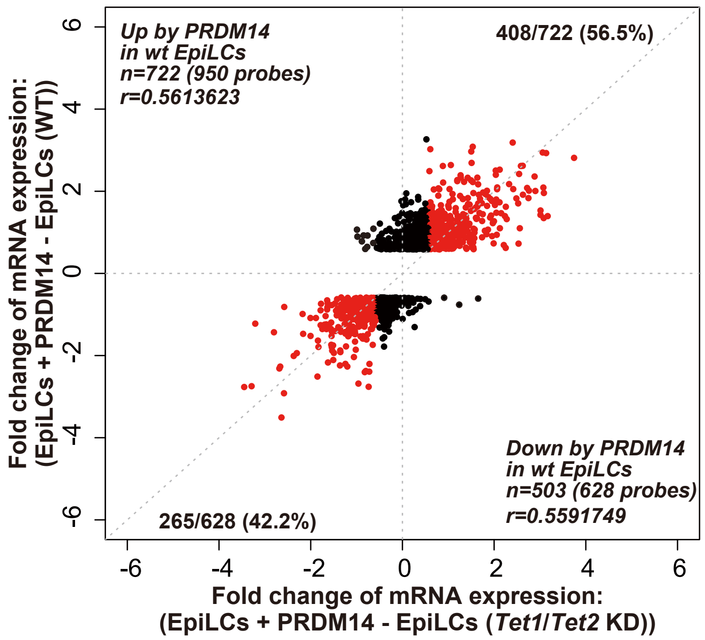

B

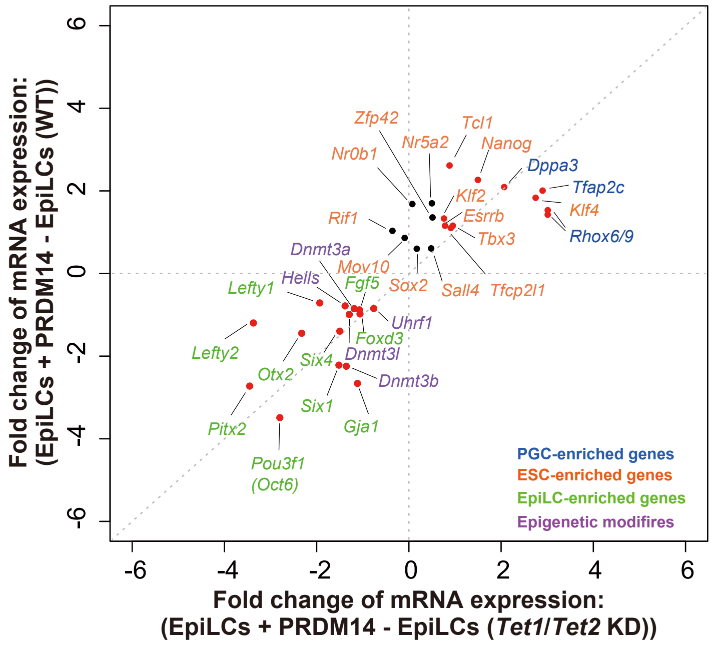

Supplementary Fig. 4 The effect of *Tet1*/*Tet2* KD in the conversion from EpiLCs to ESCLCs by PRDM14

(**A**, **B**) Scatterplot of microarray data represents genes upregulated or downregulated by PRDM14 in control and *Tet1*/*Tet2* KD EpiLCs. The y-axis indicates fold changes between + Dox control EpiLCs and –Dox control EpiLCs by applying ≥1.5-fold difference as a cut-off value. The x-axis indicates a fold change between + Dox *Tet1*/*Tet2* KD EpiLCs and –Dox *Tet1*/*Tet2* KD EpiLCs. Red dots indicate genes upregulated or downregulated by PRDM14 both in the control and *Tet1*/*Tet2* KD EpiLCs.

Supplementary Figure S5

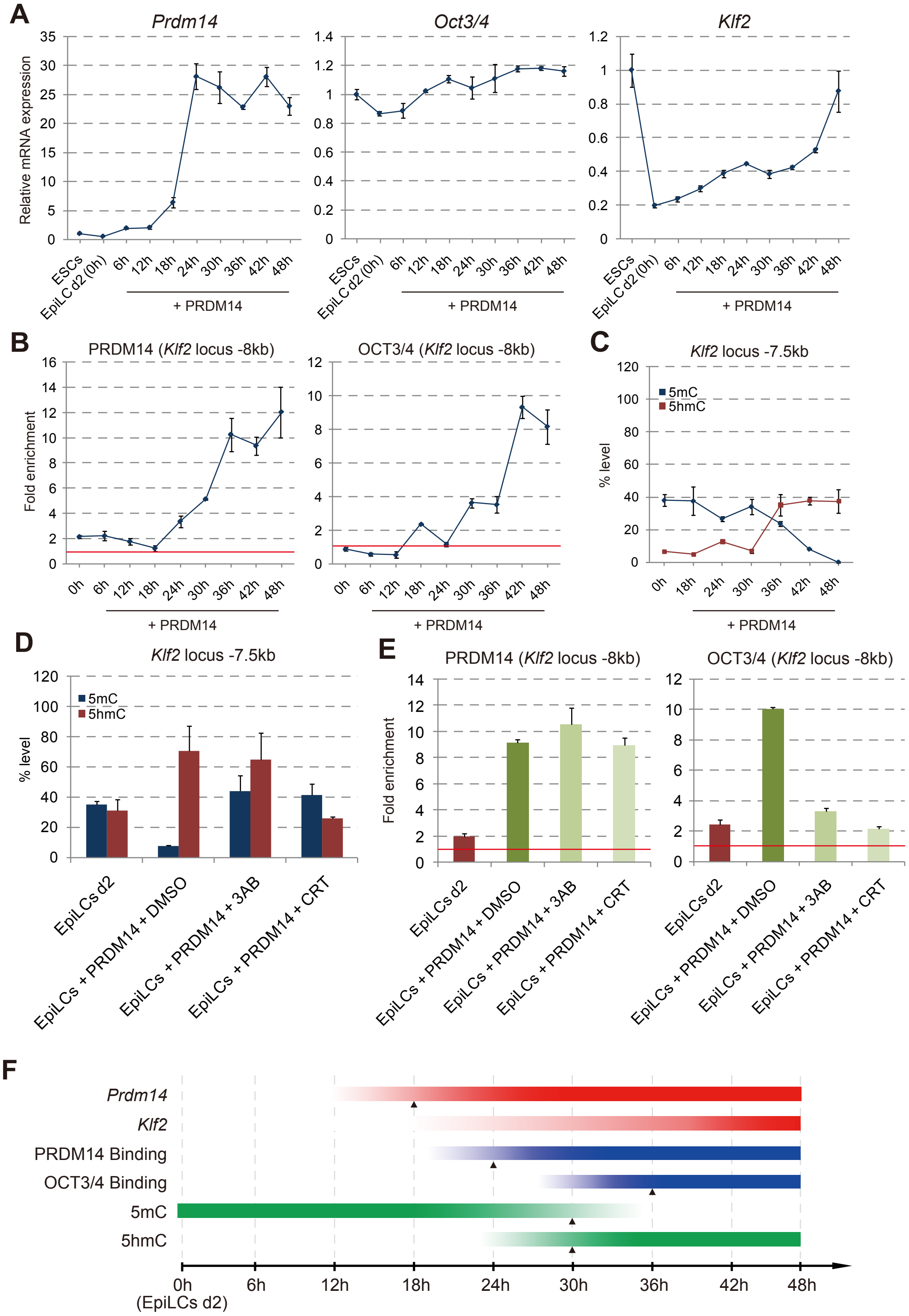

Supplementary Fig. 5 Molucular mechanism of *Klf2* activation by PRDM14

(**A**) qRT-PCR analysis of *Prdm14*, *Oct3/4* and *Klf2* in the conversion from EpiLCs to ESC-like cells by PRDM14. (**B**) ChIP analysis of PRDM14 and OCT3/4 on *Klf2* distal enhancer region in the conversion from EpiLCs to ESC-like cells by PRDM14. Fold enrichment values were calculated by comparing IgG control with PRDM14 and OCT3/4 antibody. (**C**) GlucMS-qPCR analysis of 5mC and 5hmC at *Klf2* distal enhancer region in the conversion from EpiLCs to ESC-like cells by PRDM14. (**D**) GlucMS-qPCR analysis of 5mC and 5hmC at neighboring *Klf2* distal enhancer region in EpiLCs d2 and EpiLCs d4 + PRDM14 (DMSO, 3AB, CRT). (**E**) ChIP analysis of PRDM14 and OCT3/4 on *Klf2* distal enhancer region in EpiLCs d2 and EpiLCs + PRDM14 (DMSO, 3AB, CRT). Fold enrichment values were calculated by comparing IgG control with PRDM14 and OCT3/4 antibody. Error bar indicates ± s.d. of technical duplicate of qPCR (**F**) A summary for the temporal hierarchy of events (PRDM14 and OCT3/4 binding, 5mC removal, and activation of *Klf2* expression)

Supplemental Experimental Procedures

Teratoma formation and histological analysis

Approximately 5 × 10^6 EpiLCs expressing PRDM14, cultured without LIF, were injected subcutaneously into both the flanks of female nude mice. After 30 days, the tumors were excised, embedded in paraffin, and sectioned into 4-μm-thick slices. Hematoxylin & Eosin staining was done according to the standard protocol. Markers for the three germ layers were monitored with immunofluorescence with antibodies: α-AFP (Proteintech Group, Inc.; 14550-1-AP), α-SMA (Abcam; ab5694), and α-β-Tubulin (Cell Signaling; #2146).

ChIP assay

Cells were cross-linked by adding 1% formaldehyde. The ChIP assay was carried out using the ChIP Assay kit (Upstate Biotechnology) according to the manufacturer's protocol (http://www.millipore.com/). Cross-linked protein/DNA was incubated with antibodies [α-FLAG (Sigma; F1804), α-OCT3/4 (N-19) (Santa Cruz; sc-8629)] for 12 hours at 4 °C with rotation. Antibody/protein/DNA complexes were collected by using

Dynabeads Protein A (Invitrogen) or Dynabeads Protein G (Invitrogen). De-fixed DNA was purified by phenol/chloroform extraction, followed by ethanol precipitation, and analyzed by qPCR. The primers used are presented in **Table S1**.

Expression microarray and data analysis

Total RNA was purified using the PureLink™ RNA Mini Kit (Ambion). Purified total RNA (150 ng) was labeled using a GeneChip 3' IVT Express Kit (Affymetrix) and hybridized to MG-430 PM array strip performed on the GeneAtlas system (Affymetrix), according to the manufacturer's instructions. CELL files of GenChIP data were normalized by the Robust Multi-array Average (RMA) with the default setting in Affymetrix Expression Console™. Unsupervised hierarchical clustering (UHC) with Pearson correlation coefficient and scatter plot analysis was performed by using R program. Upregulated and downregulated genes by PRDM14 in EpiLCs was clustered using average linkage hierarchical clustering with Pearson correlation distance measure and visualized in a heatmap by Gene Cluster 3.0 and Java Treeview. Gene Ontology analysis was performed using DAVID version 6.7 (http://david.abcc.ncifcrf.gov/) with

default parameters. Venn diagrams were constructed using BioVenn(Hulsen et al., 2008).

Glucosylation of genomic DNA followed by methylation-sensitive (GlucMS)-qPCR analysis

Genomic DNA (2350 ng) was treated with T4 phage β-glucosyltransferase (T4-BGT) (NEB M0357S), according to the manufacturer's instructions. Glycosylated genomic DNA (750 ng) was digested with 40 U of either Hpa II, Msp I, or no enzyme at 37 °C overnight, followed by inactivation with proteinase K treatment. The Hpa II- or Msp I-resistant fractions were quantified by qPCR using primers designed around one Hpa II/Msp I site, and normalizing to the region lacking Hpa II/Msp I sites. Resistance to Msp I directly translated to percentage of 5hmC, whereas the percentage of 5mC was calculated by subtracting the 5hmC contribution from the total Hpa II resistance. The primers used are presented in **Table S1**.

Inhibition of the base excision repair pathway

Concurrently with the induction of PRDM14, small molecule inhibitors, either 5 mM 3-AB (Sigma-Aldrich) or 100 μM CRT0044876 (Calbiochem) of 0.1% DMSO, were added to the medium in order to inhibit the base excision repair pathway.

ChIP-seq and downstream *in silico* analysis

Library for the Illumina sequencing platform was prepared from 20 ng input DNA or 1 ng ChIP DNAs using the KAPA LTP library preparation kit (Kapa Biosystems) with Illumina TruSeq-compatible multiplex adapters. Quality control check for length distribution and concentration of DNA fragments in the prepared libraries was performed using the Agilent 2100 Bioanalyzer (Agilent Technologies). The prepared ChIP DNA libraries were sequenced in Rapid Run mode of HiSeq 1500 (Illumina) for 80 nt single-end reads, with 5% PhiX spike-in.

Raw sequence data were analyzed using FastQC program ver. 0.11.1 (http://www.bioinformatics.bbsrc.ac.uk/projects/fastqc/). Trimming of adapter sequences and the removal of low-quality reads was performed with the Trim Galore ver. 0.3.3 (http://www.bioinformatics.babraham.ac.uk/projects/trim_galore/) with

parameters '-e 0.1 -q 20'. The processed reads were aligned with the mouse genome sequences mm10 by Bowtie2(Langmead and Salzberg, 2012) ver. 2.2.5 with default parameters. Peak calling for individual ChIP-DNA libraries were performed by MACS2 ver. 2.1.0.20150316(Feng et al., 2012) with the filtering parameter as '-q 0.01', using reads from input DNA library as control. To identify differentially enriched regions between two ChIP DNA libraries, the bdgdiff function of MACS2 was used. Evaluation and visualization of ChIP peaks was done by employing the UCSC Integrative Genomics Viewer (IGV) ver. 2.3.36(Thorvaldsdottir et al., 2013).

REFERENCES

Feng, J., Liu, T., Qin, B., Zhang, Y., and Liu, X.S. (2012). Identifying ChIP-seq enrichment using MACS. Nat Protoc *7*, 1728-1740.

Hulsen, T., de Vlieg, J., and Alkema, W. (2008). BioVenn - a web application for the comparison and visualization of biological lists using area-proportional Venn diagrams. BMC Genomics *9*, 488.

Langmead, B., and Salzberg, S.L. (2012). Fast gapped-read alignment with Bowtie 2. Nat Methods *9*, 357-359.

Thorvaldsdottir, H., Robinson, J.T., and Mesirov, J.P. (2013). Integrative Genomics Viewer (IGV): high-performance genomics data visualization and exploration. Brief Bioinform *14*, 178-192.
