# Supplementary material for: PRDM14 Drives OCT3/4 Recruitment via Active Demethylation in the Transition from Primed to Naive Pluripotency
Source: Stem Cell Reports. 2016 Nov 17;7(6):1072–86. doi: 10.1016/j.stemcr.2016.10.007 (PMC5161533; doi:10.1016/j.stemcr.2016.10.007)
Supplement: Document S2. Article plus Supplemental Information [file mmc3.pdf]

Stem Cell Reports
Article
OPEN ACCESS

PRDM14 Drives OCT3/4 Recruitment via Active Demethylation in the Transition from Primed to Naive Pluripotency

Naoki Okashita,[1,4] Yoshiaki Suwa,[1] Osamu Nishimura,[2] Nao Sakashita,[1] Mitsutaka Kadota,[2] Go Nagamatsu,[3] Masanori Kawaguchi,[1] Hiroki Kashida,[1] Ayaka Nakajima,[1] Makoto Tachibana,[4] and Yoshiyuki Seki[1,*]

[1]Department of Biomedical Chemistry, School of Science and Technology, Kwansei Gakuin University, 2-1, Gakuen, Sanda, Hyogo 669-1337, Japan
[2]Phyloinformatics Unit, RIKEN Center for Life Science Technologies, 2-2-3 Minatojima-minami, Kobe 650-0047, Japan
[3]Department of Stem Cell Biology and Medicine, Graduate School of Medical Science, Kyushu University, Fukuoka 812-8582, Japan
[4]Department of Enzyme Chemistry, Institute for Enzyme Research, Tokushima University, Tokushima 770-8503, Japan
*Correspondence: yseki@kwansei.ac.jp
http://dx.doi.org/10.1016/j.stemcr.2016.10.007

SUMMARY

Primordial germ cells (PGCs) are specified from epiblast cells in mice. Genes associated with naive pluripotency are repressed in the transition from inner cell mass to epiblast cells, followed by upregulation after PGC specification. However, the molecular mechanisms underlying the reactivation of pluripotency genes are poorly characterized. Here, we exploited the in vitro differentiation of epiblast-like cells (EpiLCs) from embryonic stem cells (ESCs) to elucidate the molecular and epigenetic functions of PR domain-containing 14 (PRDM14). We found that *Prdm14* overexpression in EpiLCs induced their conversion to ESC-like cells even in the absence of leukemia inhibitory factor in adherent culture. This was impaired by the loss of Kruppel-like factor 2 and ten-eleven translocation (TET) proteins. Furthermore, PRDM14 recruited OCT3/4 to the enhancer regions of naive pluripotency genes via TET-base excision repair-mediated demethylation. Our results provide evidence that PRDM14 establishes a transcriptional network for naive pluripotency via active DNA demethylation.

INTRODUCTION

Cells in the human body can be broadly classified as two major types, germline and somatic cells. The fusion of fully differentiated germ cells (i.e., sperm and oocyte) produces a totipotent zygote. Since all cells harbor the same genetic code, differentiation depends on the epigenetic state of each cell; germ cells reprogram epigenetic information during their specification, development, and maturation to acquire toti- or pluripotency. Germ cells in mice are specified from proximal epiblast cells that lack expression of some subsets of pluripotency genes such as *Nanog*, Kruppel-like factor 2 (*Klf2*), and T cell leukemia/lymphoma 1 (*Tcl1*), which are regulated by the transcription factors B lymphocyte-induced maturation protein 1 (BLIMP1), PR domain-containing 14 (PRDM14), and transcription factor AP-2 gamma (TFAP2C) (Nakaki et al., 2013; Ohinata et al., 2005, 2009; Weber et al., 2010; Yabuta et al., 2006; Yamaji et al., 2008). Specified PGCs reactivate pluripotency-associated genes and can be used to derive embryonic germ cells (EGCs) via stimulation with three cytokines, basic fibroblast growth factor (bFGF), stem cell factor (SCF), and leukemia inhibitory factor (LIF) (Matsui et al., 1992). Migrating PGCs in the hindgut replace repressive DNA methylation and H3K9 methylation marks with a repressive H3K27 trimethylation mark in their genome during migration (Seki et al., 2005, 2007). DNA demethylation occurs as a two-step process: the first demethylation wave occurs in a genome-wide manner during migration and a second wave occurs in a locus-specific manner, for example at germline-specific genes and imprinted loci at the time when PGCs arrive at the gonads (Seisenberger et al., 2012). CpG methylation marks can be removed via replication-dependent and independent mechanisms (Wu and Zhang, 2010). The former is regulated by inhibition of DNA methyltransferase activity during de novo DNA synthesis, whereas the latter (also known as active demethylation) is triggered by oxidation of 5-methylcytosine catalyzed by ten-eleven translocation (TET) proteins, which is followed by base excision repair (BER) (Hackett et al., 2012). PGCs use both active and passive demethylation to erase genome-wide methylation marks (Hajkova et al., 2008; Kagiwada et al., 2013; Kawasaki et al., 2014; Ohno et al., 2013; Popp et al., 2010). Global hypomethylation is induced in the transition from metastable embryonic stem cells (ESCs) cultured with serum plus LIF to ground-state ESCs cultured with 2i plus LIF, which is similar to the first wave of DNA demethylation in migratory PGCs (Ficz et al., 2013).

We have previously shown that PRDM14 is transiently expressed in the inner cell mass (ICM) of blastocysts at embryonic day 3.5 (E3.5) and reactivated in specifying PGCs at approximately E6.25 (Yamaji et al., 2008). Knockout (KO) studies have demonstrated that expression of *Prdm14* in the ICM is required for priming of the epiblast cells for proper somatic differentiation (Payer et al., 2013), and that expression of PGCs is critical for establishment of the germline in mice (Yamaji et al., 2008). Furthermore, induction of PRDM14 in epiblast-like cells (EpiLCs) induced from ESCs is sufficient for PGC-like cell (PGCLC) induction

 © 2016 The Author(s).
This is an open access article under the CC BY license (http://creativecommons.org/licenses/by/4.0/).

in aggregate culture (Hayashi et al., 2011). KO and overexpression studies of *Prdm14* suggest that PRDM14 is a critical determinant for PGC specification in mice. However, recent studies regarding human PGC induction from pluripotent cells have clarified a difference in expression of *Prdm14* between mice and humans during the transition from pluripotent cells to PGCs (Irie et al., 2015; Sasaki et al., 2015). Interestingly, *PRDM14* is consistently expressed at moderate levels in pluripotent cells, i.e., primitive streak cells, which are precursors of PGCs in human, and PGCs, suggesting that PRDM14 is not a critical determinant for PGC specification in humans.

Here, we exploited adherent culture of EpiLCs in which *Prdm14* expression is controlled by doxycycline (Dox) to reveal the molecular and epigenetic function of PRDM14 during the transition from epiblast cells to PGCs in mice. We showed that inducing *Prdm14* expression in EpiLCs in adherent culture promotes pluripotency and self-renewal, resulting in conversion from EpiLCs to ESC-like cells (ESCLCs).

RESULTS

PRDM14 Overexpression Converts EpiLCs to ESC-like Cells

Prdm14 is expressed in proximal epiblast cells at around E6.5 that constitute PGC precursors in mice. In three-dimensional aggregate culture of EpiLCs, PRDM14 has been shown to be sufficient to induce PGCLCs (Nakaki et al., 2013). To exclude the effect of cell-cell contact in three dimensions and investigate the direct function of PRDM14 in PGC development, we exploited adherent culture of EpiLCs, in which *Prdm14* expression was controlled by Dox treatment (ROSA-Tet-off [Figures 1 and 5] and piggyBac Tet-on [Figures 2, 3, 4, and 6]) (Figures 1A and 1B) (Hayashi et al., 2011). ESCs were transferred onto fibronectin and stimulated with bFGF and activin A in N2B27 medium containing 1% KO serum replacement (KSR), and 2 days after EpiLC induction, *Prdm14* expression was induced by removal of Dox in Glasgow minimum essential medium containing 15% KSR (GK15), which is the basic medium used for PGCLC induction (Figures 1C and 1D) (Ohinata et al., 2009). The morphology of EpiLCs expressing *Prdm14* changed from flattened to small, compact colonies, and the cells regained alkaline phosphatase (AP) activity to a degree similar to that of ESCs, which was associated with the reactivation of pluripotency genes (Figures 1E and 1F). Interestingly, the expression of early PGC markers in EpiLCs expressing PRDM14 was comparable with that in ESCs, and we were able to expand EpiLCs expressing PRDM14 at multiple passages, which clearly indicates that PRDM14 induction in EpiLCs does not induce PGCLCs (Figure S1A). To evaluate the self-renewal capacity and pluripotency of *Prdm14*-expressing EpiLCs, we assessed colony formation along with AP activity in ESCs grown at low density under standard culture conditions (serum plus LIF). The number of AP-positive colonies was higher for EpiLCs overexpressing PRDM14 than for control EpiLCs (Figure 1G). Interestingly, despite the absence of LIF, we were able to expand PRDM14-expressing EpiLCs several times, and these cells retained expression of pluripotency genes except for *Klf4*, which is a direct target of LIF signaling (Figure S1B). Moreover, the cells formed three germ layers in the subcutaneous region of nude mice, indicating that PRDM14 expression confers self-renewal capacity and pluripotency in EpiLCs independent of LIF signaling (Figure 1H).

To determine the transcriptional dynamics in PRDM14-expressing EpiLCs, we performed a microarray analysis using total RNA from ESCs, EpiLCs at day 2 (d2), EpiLCs + PRDM14, and EpiLCs (d4). Unsupervised hierarchical clustering revealed that the global gene expression profile of EpiLCs + PRDM14 was more similar to that of ESCs than that of EpiLCs (Figure 2A). Scatterplots of EpiLCs (d2) and EpiLCs + PRDM14 showed that 669 genes, including those associated with pluripotency, were upregulated, and 480 genes, including those encoding DNA methyltransferases or associated with differentiation, were downregulated by *Prdm14* expression in EpiLCs (Figure 2B). Unsupervised hierarchical clustering of genes up- or downregulated by PRDM14 in EpiLCs formed four clusters (Figure 2C). Genes in cluster 1 did not show a significant change in the transition from ESCs to EpiLCs, but were upregulated thereafter, whereas cluster-2 genes showed downregulation from ESCs to EpiLCs, followed by upregulation. Genes in cluster 3 showed relatively high expression from ESCs to EpiLCs followed by downregulation, whereas those in cluster 4 showed upregulation from ESCs to EpiLCs before subsequent downregulation. According to gene ontology (GO) functional annotations, stem cell maintenance (e.g., *Tcl1*, *Nanog*, *nuclear receptor subfamily* [*Nr*]*5a2*, and estrogen-related receptor β [*Esrrb*]), and DNA methylation (e.g., *Dnmt3a/b*) genes were enriched in clusters 2 and 4, respectively (Figures 2C and 2D). PRDM14 overexpression in epiblast stem cells (EpiSCs) partially activated pluripotency genes without inducing their conversion to ESCs, even in the presence of LIF (Gillich et al., 2012). To identify candidate genes regulated by PRDM14 that can account for the difference between EpiLC and EpiSC conversion to ESCs, we compared the top 1,000 probes that were differentially upregulated by PRDM14 overexpression in EpiLCs and EpiSCs according to published data (Gillich et al., 2012). Critical pluripotency factors such as *Tcl1*, *Nanog*, *Nr5a2*, transcription factor CP2-like 1 (*Tfcp2l1*), and *T-box 3* (*Tbx3*) were upregulated by PRDM14 in EpiLCs but not in EpiSCs (Figure 2E), suggesting that differences in the

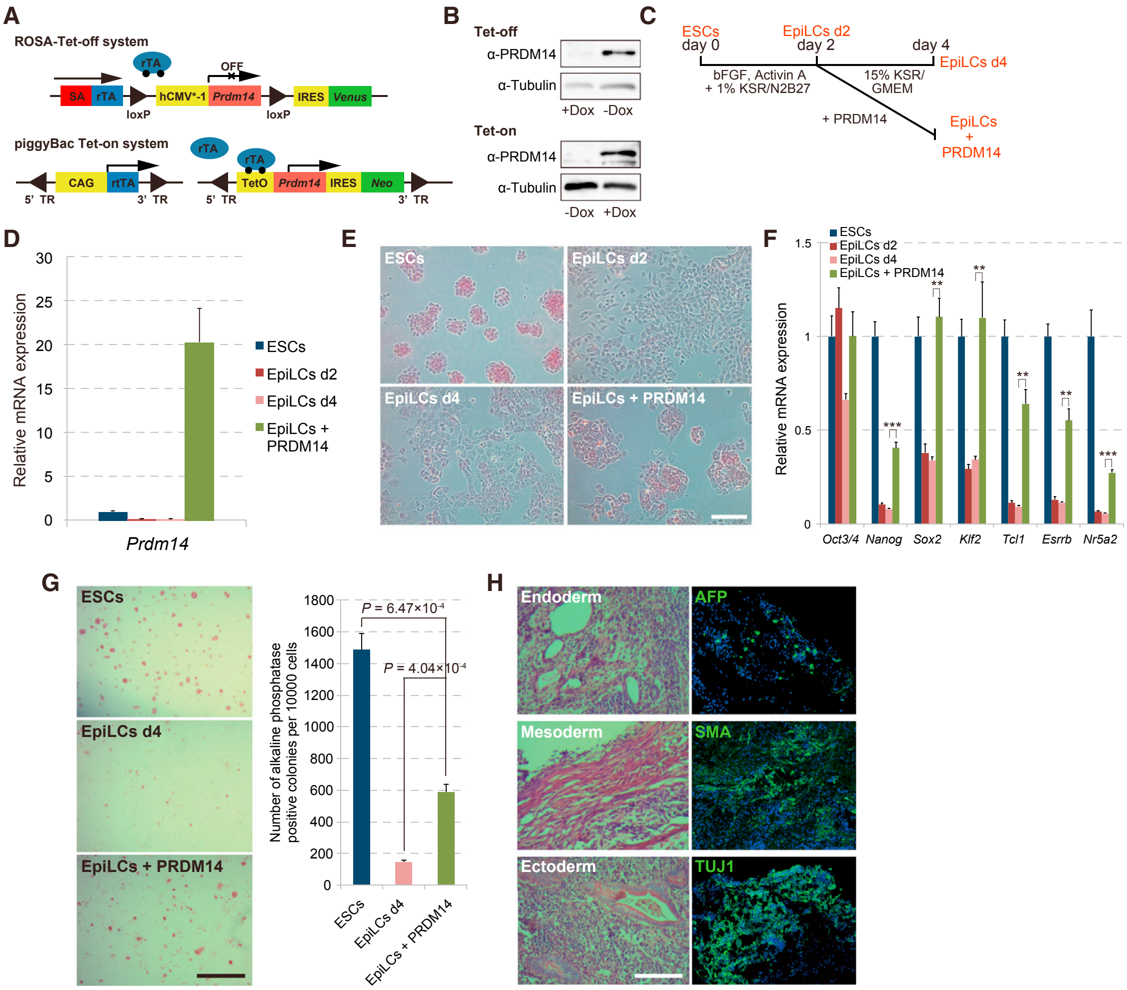

Figure 1. PRDM14 Induces the Transition of EpiLCs to ESCLCs

(A) Constructs of the ROSA-Tet-off system and piggyBac Tet-on system.

(B) Western blotting of PRDM14 and tubulin.

(C) Scheme of the transition of ESCs to EpiLCs and of EpiLCs to ESCLCs.

(D) qRT-PCR analysis of *Prdm14* expression in ESCs, EpiLCs (d2), EpiLCs (d4), and EpiLCs + PRDM14. Error bars indicate ±SD of a biological triplicate.

(E) AP staining of ESCs, EpiLCs (d2), EpiLCs (d4), and EpiLCs + PRDM14. Scale bar, 50 μm.

(F) qRT-PCR analysis of pluripotency genes in ESCs, EpiLCs (d2), EpiLCs (d4), and EpiLCs + PRDM14. Error bars indicate ±SD of a biological triplicate.

(G) Colony formation by ESCs, EpiLCs (d4), and EpiLCs + PRDM14 was assessed by AP staining over 3 days in the presence of serum + LIF. Error bars indicate ±SD of biological triplicates. p Values were calculated with the Student's t test. Scale bar, 200 μm.

(H) (Left) Teratoma formation by EpiLCs expressing *Prdm14* cultured in the absence of LIF. (Right) Immunofluorescent staining of α-fetoprotein (AFP), smooth muscle actin (SMA), and class III β-tubulin (TUJ1) in teratoma sections. Scale bar, 200 μm.

$^{**}p < 0.01$, $^{***}p < 0.001$, Student's t test.

epigenetic landscape of pluripotency-associated genes between EpiLCs and EpiSCs may be critical for their activation by PRDM14. In the case of genes downregulated by PRDM14, epigenetic modifiers controlling DNA and H3K9 methylation—including *Dnmt3a*, *Dnmt3l*, *ubiquitin-like PHD* and *RING finger domain-containing protein 1* (*Uhrf1*), *helicase lymphoid-specific* (*Hells*), and *suppressor of variegation 3–9 homolog 1* (*Suv39h1*)—were downregulated by PRDM14 only in EpiLCs (Figure S2A), which may be necessary for the establishment of an epigenetic background that ensures the conversion from EpiLCs to ESCLCs.

We used adherent EpiLC cultures to investigate the function of PRDM14 while excluding the effects of

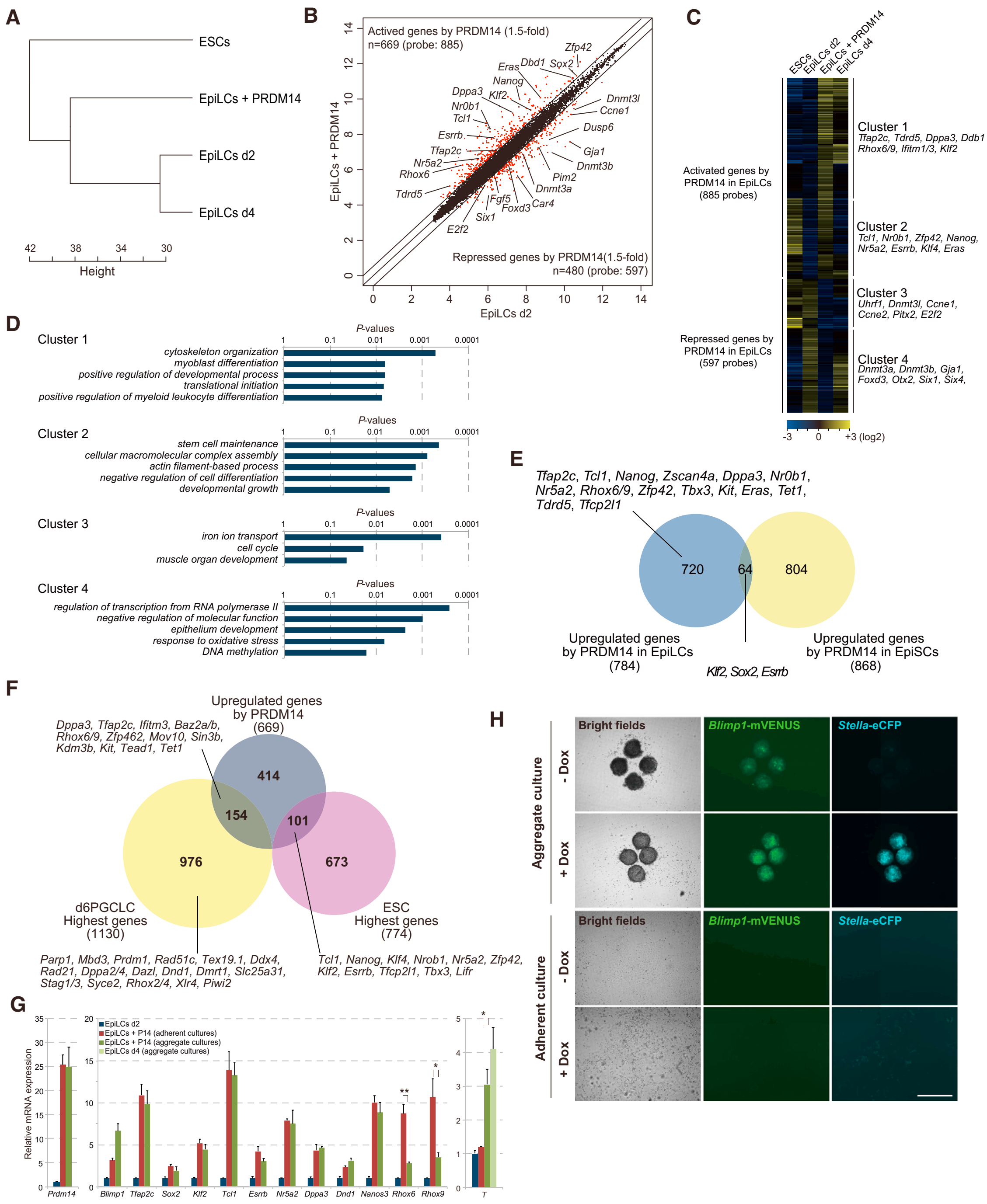

Figure 2. Global Gene Expression Profiles in the Transition from EpiLCs to ESCLCs

(A) Unsupervised hierarchical cluster analysis of ESCs, EpiLCs (d2), EpiLCs (d4), and EpiLCs + PRDM14.

(B) Scatter plots comparing the transcriptomes of EpiLCs + PRDM14 and EpiLCs (d2).

(C) Heatmap visualization of data obtained by unsupervised hierarchical cluster analysis of genes up- and downregulated by PRDM14 in EpiLCs.

(D) GO term overrepresentation analysis using DAVID.

(E) Comparison of genes upregulated by PRDM14 in EpiLCs versus EpiSCs.

(F) Comparison of genes upregulated by PRDM14 and those most highly expressed in ESCs and d6 PGCLCs (Kurimoto et al., 2015).

(legend continued on next page)

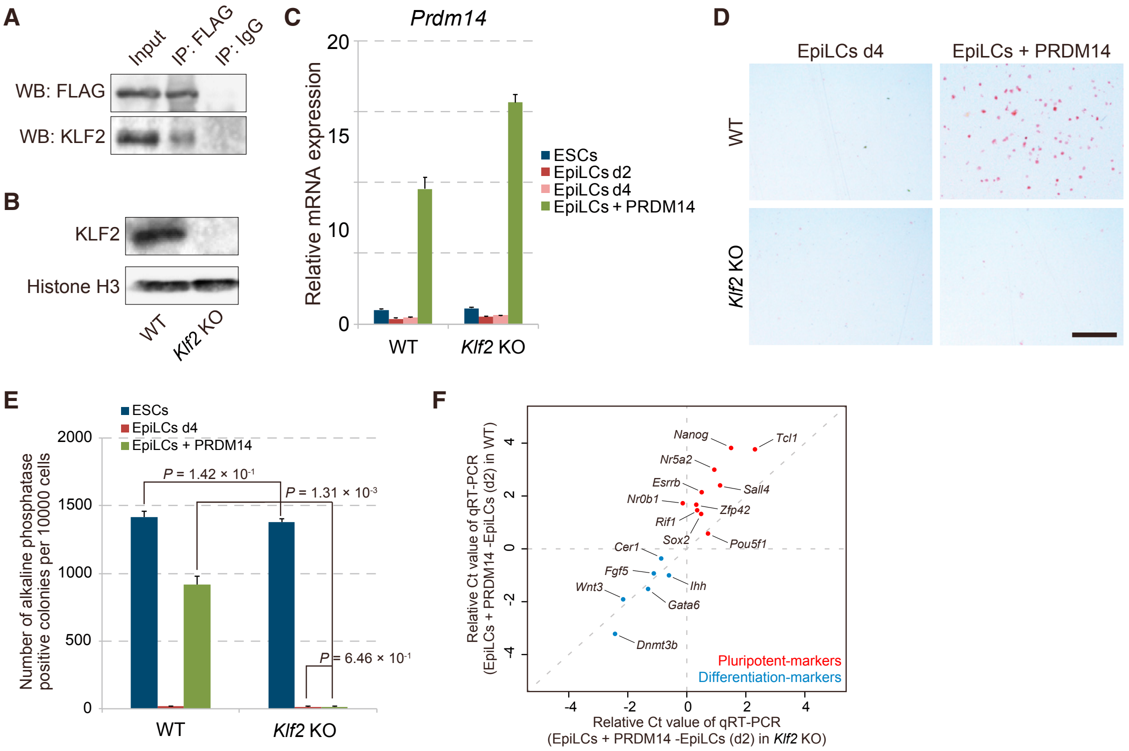

Figure 3. *Klf2* Is Required for the PRDM14-Induced Reversion of EpiLCs to ESCLCs

(A) Immunoprecipitation of PRDM14 with KLF2 in ESCs.

(B) Western blot analysis of KLF2 expression in WT and *Klf2* KO ESCs.

(C) qRT-PCR analysis of *Prdm14* expression in WT and *Klf2* KO ESCs. Error bars indicate ±SD of a biological triplicate.

(D) Colony formation by EpiLCs with or without *Prdm14* induction induced from WT or *Klf2* KO ESCs, as assessed by AP staining. Scale bar, 200 μm.

(E) Quantification of colony formation shown in (D). Error bars indicate ±SD of biological triplicates. p Values were calculated with Student's t test.

(F) Scatter plot shows relative Ct values of pluripotency-associated genes (red dots) and differentiation markers (blue dots) determined by qRT-PCR. The x axis indicates relative Ct values of each gene (EpiLCs + PRDM14 − EpiLCs [d2]) in WT, and the y axis indicates relative Ct values of each gene (EpiLCs + PRDM14 − EpiLCs [d2]) in *Klf2* KO.

three-dimensional cell-cell contact. EpiLCs expressing PRDM14 could be expanded for long time periods, implying that EpiLCs were not induced to become PGCLCs under our culture conditions. In contrast, forced expression of PRDM14 in aggregate EpiLC cultures induced PGCLCs but not ESCLCs (Nakaki et al., 2013). Germ cell specification consists of two major events: the acquisition of potential pluripotency (by reactivation of genes such as *Nanog*, *Klf2*, and *Tcl1*) and of germ cell character (by activation of *Blimp1*, *Nanos3*, and *dead-end protein homology 1*) (Kurimoto et al., 2008; Yamaji et al., 2008). The difference in cell fate between PRDM14-expressing EpiLCs grown as adherent or aggregate cultures suggests that acquisition of pluripotency is directly controlled by PRDM14, while three-dimensional cell-cell interactions between EpiLCs are required for the induction of germ cell characteristics, including the activation of germline-specific genes. A recent analysis of global gene expression in the differentiation of ESCs to EpiLCs and of EpiLCs to PGCLCs identified genes that were enriched in ESCs, EpiLCs, and d2 and d6 PGCLCs (Kurimoto et al., 2015). We compared the characteristics of EpiLCs expressing PRDM14 grown under our

(G) qRT-PCR analysis of pluripotency and germ cell-related genes in EpiLCs, and EpiLCs expressing PRDM14 and grown as adherent or aggregate cultures. Error bars indicate ±SD of a biological triplicate. *$p < 0.05$, **$p < 0.01$, Student's t test.

(H) Comparison of the expression of PGC markers, Blimp1 (mVENUS) and stella (eCFP), between adherent culture and aggregate culture of EpiLCs with PRDM14. Scale bar, 200 μm.

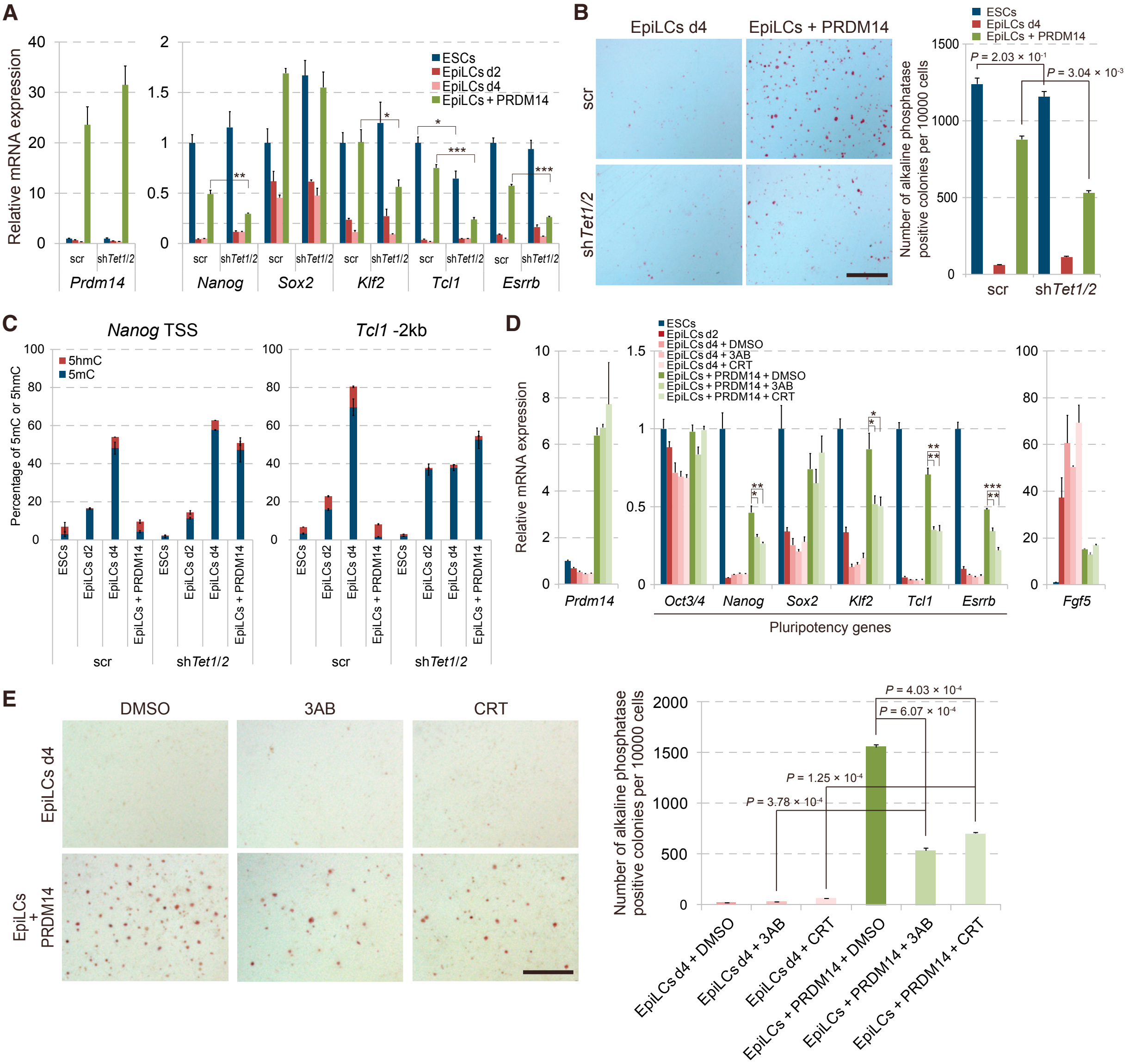

Figure 4. Reversion from Primed to Naive Pluripotency Induced by PRDM14 via the TET-BER Pathway

(A) qRT-PCR analysis of *Prdm14* and pluripotency genes in ESCs, EpiLCs (d2), EpiLCs (d4), and EpiLCs + PRDM14 in WT and *Tet1/2* KD backgrounds. Error bars indicate ±SD of a biological triplicate. *p < 0.05, **p < 0.01, ***p < 0.001, Student's t test.

(B) Colony formation by EpiLCs with or without *Prdm14* induction induced from WT and *Tet1/2* KD ESCs, as determined by AP staining. Scale bar, 200 μm. Error bars indicate ±SD of biological triplicates. p Values were calculated with Student's t test.

(C) Glucosylation of genomic DNA followed by methylation-sensitive (GlucMS)-qPCR analysis showing the percentage of 5mC and 5hmC marks in or near PRDM14 binding regions of the *Nanog* and *Tcl1* enhancers in ESCs, EpiLCs (d2), EpiLCs (d4), and EpiLCs + PRDM14 in WT and *Tet1/2* KD backgrounds. Error bars indicate ±SD of a biological triplicate.

(D) qRT-PCR analysis of *Prdm14*, pluripotency genes, and *Fgf5* in ESCs, EpiLCs (d2), EpiLCs (d4), and EpiLCs + PRDM14 treated with DMSO (control), 3-aminobenzamide (3AB), and CRT 004876 (CRT). Error bars indicate ±SD of a biological triplicate. *p < 0.05, **p < 0.01, ***p < 0.001, Student's t test.

(E) Colony formation by EpiLCs + PRDM14 treated with DMSO, 3AB, and CRT as assessed by AP staining. Error bars indicate ±SD of a biological triplicate. p Values were calculated with Student's t test. Scale bar, 200 μm.

culture conditions with ESC highest genes, d6 PGCLCs, and EpiLC highest genes in a Venn diagram. The majority of transcription factors critical for ESC pluripotency among ESC highest genes were upregulated by PRDM14 in adherent EpiLC cultures, while genes most highly expressed in d6 PGCLCs were generally not upregulated by

PRDM14 in these cultures including *Blimp1* (*Prdm1*), a critical determinant of germ cell specification (Ohinata et al., 2005) (Figures 2F and 2G). These findings led us to speculate that a combination of *Prdm14* induction and three-dimensional cell-cell contact is necessary for the induction of *Blimp1* expression in EpiLCs. To clarify the response of EpiLCs to PRDM14 induction in aggregate culture and adherent culture, we established PRDM14-inducible ESCs carrying two reporters, *Blimp1-mVENUS* (BV) and *stella-eCFP* (SC) (BVSC), and monitored the fluorescence of mVENUS and eCFP in different culture conditions (Hayashi et al., 2011). At 2 days after PRDM14 induction in EpiLCs at aggregate culture, the aggregates showed both BV and SC expression (Figure 2H), which provides evidence of PGCLC induction, as shown in a previous study (Nakaki et al., 2013). By contrast, adherent-cultured EpiLCs expressing PRDM14 showed no BV expression and weak SC expression, which is consistent with the expression profile in ESCs (Hayashi et al., 2011). *Blimp1* is upregulated by the mesoderm marker gene *T* (*Brachyury*) (Aramaki et al., 2013). We therefore compared *T* expression levels in adherent and aggregate EpiLC cultures. Interestingly, *T* was only upregulated in EpiLC aggregate cultures both with and without *Prdm14* induction (Figure 2G), suggesting that three-dimensional cell-cell interactions are required for *T* expression, which induces PGCs along with PRDM14. A comparison of genes downregulated by PRDM14 in adherent EpiLC cultures with EpiLC highest genes showed that many EpiLC highest genes including *Otx2* and *Foxd3* (Buecker et al., 2014; Respuela et al., 2016), which are known to important for the transition from naive to primed pluripotency, were repressed by PRDM14, indicating that PRDM14 suppresses epiblast characteristics (Figure S2B).

Klf2 Is Required for the Acquisition of Pluripotency by PRDM14

Adherent culture of EpiLCs with PRDM14 is a powerful tool to uncover the molecular and epigenetic mechanisms for potential re-establishment of pluripotency by PRDM14, because this system induces the conversion from EpiLCs to ESCLCs at high efficiency and uniformly. We first focused on the function of KLF2, which is activated soon after induction of PRDM14 in EpiLCs in adherent culture. KLF2 has been shown to act synergistically with PRDM14 in the reversion of EpiSCs to ESCs (Gillich et al., 2012); the interaction between the two proteins in ESCs was confirmed by immunoprecipitation (IP) (Figure 3A). To clarify the role of *Klf2* in the PRDM14-associated acquisition of pluripotency, we generated *Klf2* KO ESCs harboring a Dox-inducible *Prdm14* expression cassette using the clustered regularly interspaced short palindromic repeats (CRISPR)/Cas9 system (Figures 3B and 3C). Interestingly, we did not observe AP-positive colonies in *Klf2*-null EpiLCs expressing PRDM14, which demonstrated that *Klf2* is essential for the PRDM14-induced conversion of EpiLCs to ESCLCs (Figure 3D and 3E). To clarify the cause of the loss of AP activity in EpiLCs + PRDM14 in *Klf2* KO, we compared the repression of differentiation markers and activation of pluripotency-associated markers by PRDM14 in wild-type (WT) and *Klf2* KO using qRT-PCR analysis. We plotted the relative Ct values of qRT-PCR data (EpiLCs + PRDM14 − EpiLCs [d2]) for WT (y axis) and *Klf2* KO (x axis). Consistent impairment of activation of pluripotent-associated genes by PRDM14 was observed, whereas the repression of differentiation markers by PRDM14 was relatively maintained in *Klf2* KO EpiLCs (Figure 3F). These results indicate that PRDM14 cooperates with KLF2 to convert EpiLCs to ESCLCs through the activation of pluripotency-associated genes.

PRDM14-Induced Acquisition of Pluripotency Occurs via TET-Dependent Active Demethylation

We previously showed that PRDM14 promotes active demethylation of pluripotency genes in ESCs via the TET-BER pathway (Okashita et al., 2014). To assess the role of the TET-BER pathway in PRDM14-induced acquisition of pluripotency, we established a *Tet1*/*Tet2* knockdown (KD) ESC line with a Dox-inducible *Prdm14* cassette (Figure S3A). *Tet1*/*Tet2* KD prevented the reactivation of *Nanog*, *Klf2*, *Tcl1*, and *Esrrb* but not of *Sox2* and also prevented AP-positive colony formation by PRDM14 under ESC culture conditions (Figures 4A and 4B), while the repression of *Dnmt3b* and differentiation markers by PRDM14 occurred normally in *Tet1*/*Tet2* KD EpiLCs (Figure S3B). CpG methylation level at PRDM14 binding regions of *Nanog* and *Tcl1* in ESCs substantially increased in the transition from ESCs to EpiLCs; these marks were rapidly removed by *Prdm14* induction (Figure 4C). In contrast, the removal of 5-methylcytosine (5mC) by PRDM14 at *Nanog* and *Tcl1* loci was impaired by *Tet1*/*Tet2* KD. Furthermore, treatment of EpiLCs expressing PRDM14 with pharmacological inhibitors of the BER components DNA endonuclease apurinic/apyrimidinic endonuclease (3-aminobenzamide) and poly(ADP-ribose) polymerase (CRT 004876) abrogated the reversion of EpiLCs to ESCLCs, similar to the *Tet1*/*Tet2* KD phenotype (Figures 4D and 4E). To investigate the role of TET proteins in global gene expression profiles regulated by PRDM14 in EpiLCs, we compared genes that were up- or downregulated by PRDM14 in WT and *Tet1*/*Tet2* KD EpiLCs by microarray analysis. Genes that were up- or downregulated by PRDM14 in WT EpiLCs showed corresponding up- and downregulation in *Tet1*/*Tet2* KD EpiLCs (Figure S4A). These genes were of four types: ESC-, PGC-, or EpiLC-enriched, or epigenetic modifiers. We examined the effect of *Tet1*/*Tet2* KD on the transcriptional regulation

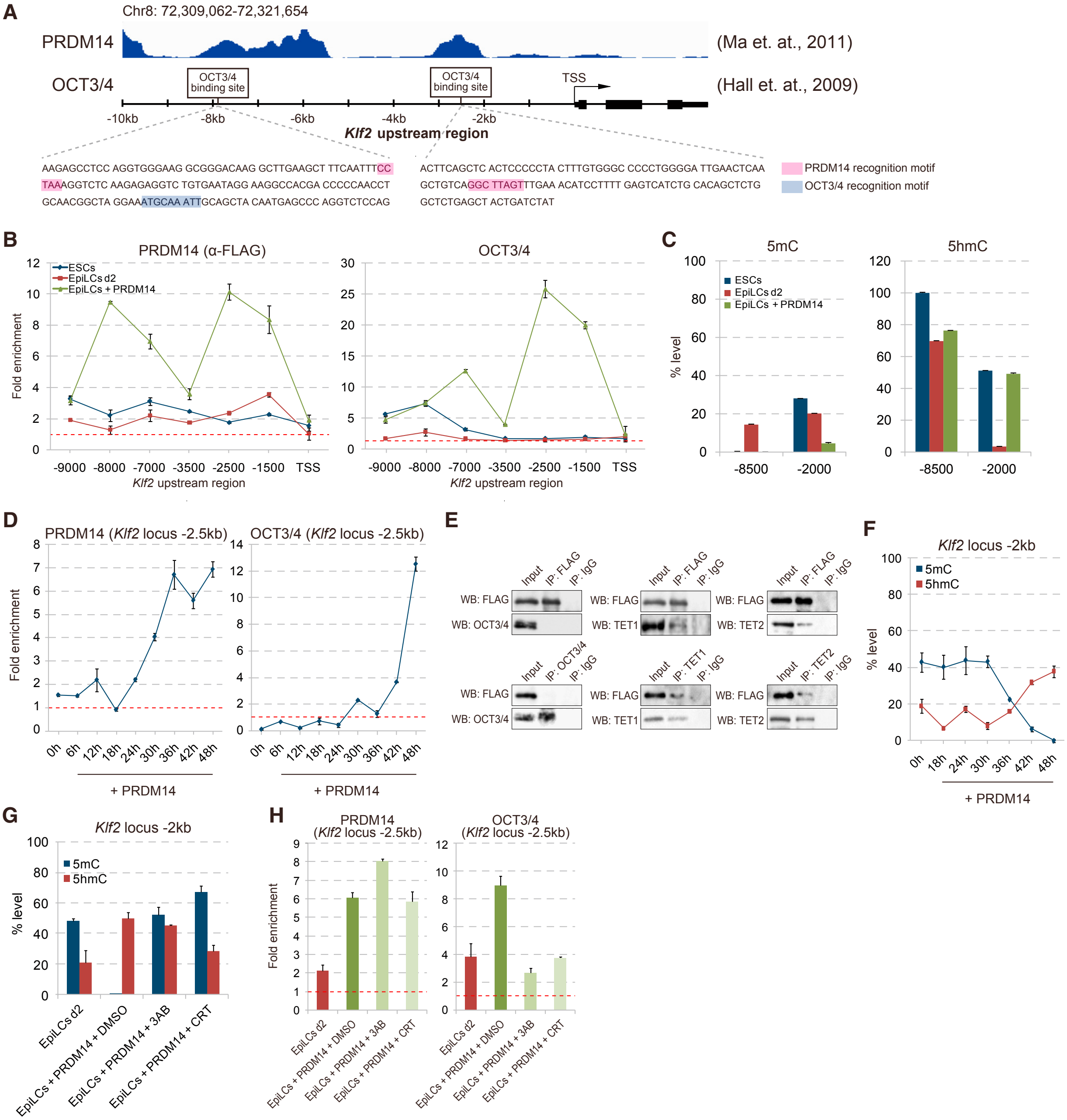

Figure 5. PRDM14 Increases OCT3/4 Recruitment to *Klf2* via the TET-BER Pathway

(A) Schematic representation of ChIP data for PRDM14 and OCT3/4 at the *Klf2* locus in ESCs (Hall et al., 2009; Ma et al., 2011) and PRDM14 and OCT3/4 recognition motifs at two enhancers.

(B) ChIP-qPCR analysis of PRDM14 (α-FLAG) and OCT3/4 at the *Klf2* locus in ESCs, EpiLCs (d2), and EpiLCs + PRDM14. Fold enrichment was calculated by comparing pre-immune immunoglobulin G (IgG) control (red dotted line) with each antibody. Error bars indicate ±SD of a technical duplicate.

(C) GlucMS-qPCR analysis showing the percentage of 5mC and 5hmC marks at two *Klf2* enhancers in ESCs, EpiLCs (d2), and EpiLCs + PRDM14. Error bars indicate ±SD of a technical duplicate.

(D) ChIP-qPCR analysis of PRDM14 (α-FLAG) and OCT3/4 at the *Klf2* locus every 6 hr after *Prdm14* induction. Error bars indicate ±SD of a technical duplicate.

(E) Western blot (WB) analysis of FLAG, OCT3/4, TET1, and TET2 levels following immunoprecipitation (IP) of EpiLCs + PRDM14 precipitated with an anti-FLAG antibody.

(legend continued on next page)

of these genes by PRDM14 in EpiLCs (Figure S4B). The expression of PGC- and EpiLC-enriched genes and epigenetic modifiers was unaltered by PRDM14 in *Tet1*/*Tet2* KD EpiLCs. In contrast, activation of ESC-enriched genes by PRDM14 was impaired in *Tet1*/*Tet2* KD EpiLCs. We observed, however, that ESC-enriched genes tended to be upregulated by PRDM14 even in *Tet1*/*Tet2* KD EpiLCs, suggesting that TET proteins cooperate with an unknown pathway regulated by PRDM14 to activate ESC-enriched genes in EpiLCs (Figure S4B).

PRDM14 Enhances OCT3/4 Binding to *Klf2* Enhancers via the TET-BER Pathway

Because *Klf2* activation is critical for acquisition of pluripotency by PRDM14, we investigated the molecular and epigenetic mechanisms of *Klf2* activation by PRDM14. Both PRDM14 and OCT3/4 bind to proximal and distal enhancers of the *Klf2* locus in ESCs (Figure 5A) (Hall et al., 2009; Ma et al., 2011). We therefore evaluated the binding of PRDM14 and OCT3/4 to the *Klf2* locus in the transition from ESCs to EpiLCs and dedifferentiation of EpiLCs to ESCLCs by chromatin IP (ChIP)-qPCR. Since ChIP-grade anti-PRDM14 antibody was unavailable, we detected the binding of exogenous FLAG-PRDM14 in EpiLCs using an anti-FLAG antibody (Figure 5B). After *Prdm14* expression was induced in EpiLCs, two distinct peaks appeared at the proximal and distal enhancers of *Klf2* (Figure 5B). Under our culture conditions, OCT3/4 was present only at distal enhancers of *Klf2*, and this peak disappeared in the transition from ESCs to EpiLCs (Figure 5B). Interestingly, induction of *Prdm14* expression enhanced the binding of OCT3/4 at both proximal and distal enhancers of *Klf2* in EpiLCs.

We next measured 5mC and 5hmC levels at two enhancers of *Klf2* in ESCs, EpiLCs, and EpiLCs + PRDM14. In ESCs, 5mC was detected only at proximal enhancers of *Klf2*, which was correlated with absence of OCT3/4 at the proximal enhancer (Figures 5B and 5C). At the distal enhancer of *Klf2*, 5mC level was transiently upregulated during the differentiation of ESCs to EpiLCs; this was followed by a downregulation induced by PRDM14. In contrast, the 5hmC level remained constant. To determine the temporal hierarchy of events (PRDM14 and OCT3/4 binding, 5mC removal, and activation of *Klf2* expression), we analyzed *Prdm14* and *Klf2* expression as well as PRDM14 and OCT3/4 binding, and 5mC and 5hmC distribution at the two *Klf2* enhancers every 6 hr after *Prdm14* induction in EpiLCs. *Prdm14* expression was detected starting 18 hr after induction; this was followed by increased PRDM14 binding at the two enhancers at 24 hr (Figures 5D, S5A, and S5B). In contrast, OCT3/4 binding at these sites first appeared 42 hr after *Prdm14* induction (Figures 5D and S5B). PRDM14 was found to interact with TET1 and TET2 but not with OCT3/4 in EpiLCs (Figure 5E), suggesting that PRDM14 indirectly enhances OCT3/4 recruitment to these enhancers. Interestingly, the level of 5mC rapidly reduced while that of 5hmC increased after PRDM14 recruitment to these enhancers (Figures 5F and S5C). To determine whether active demethylation by PRDM14 is required for OCT3/4 binding, we examined the effects of BER inhibitor (BERi) on 5mC removal from and OCT3/4 recruitment to these enhancers. BERi treatment completely disrupted the rapid removal of 5mC by PRDM14 at both *Klf2* enhancers in EpiLCs (Figures 5G and S5D). Under these conditions, OCT3/4 recruitment was abrogated although PRDM14 enrichment was unaltered (Figures 5H and S5E). Taken together, these data indicate that PRDM14 activates *Klf2* expression via active demethylation-mediated recruitment of OCT3/4 to the enhancers (Figure S5F).

To identify the genomic regions at which PRDM14 enhances OCT3/4 recruitment, we performed ChIP for OCT3/4 and PRDM14 (α-FLAG) followed by deep sequencing (ChIP-seq) in ESCs, EpiLCs, and EpiLCs expressing PRDM14, and identified 18,093, 8,136, and 13,882 OCT3/4 peaks, respectively, using the MACS2 callpeak (Feng et al., 2012). Next, to investigate the effects of PRDM14 on OCT3/4 binding, we compared the enrichment of OCT3/4 peaks among ESCs, EpiLCs, and EpiLCs expressing PRDM14 by using the MACS2 bdgdiff module (Feng et al., 2012). Over half of the OCT3/4 binding in ESCs was reduced and only 312 peaks increased in the transition of ESCs to EpiLCs (Figure 6A); 2 days after PRDM14 induction, 2,542 OCT3/4 binding peaks increased or had appeared, and those corresponding to 1,559 genes colocalized with PRDM14 binding peaks (Figure 6B). Approximately 6.3% (98 of 1,559) of genes in which OCT3/4 binding was enhanced by PRDM14 were upregulated by PRDM14, whereas approximately 2.8% (43 of 1,559) of genes in which OCT3/4 binding was upregulated by

(F) GlucMS-qPCR analysis of the percentage of 5mC and 5hmC marks at the *Klf2* locus every 6 hr after *Prdm14* induction. Error bars indicate ±SD of a technical duplicate.
(G) GlucMS-qPCR showing the percentage of 5mC and 5hmC marks at the *Klf2* locus in EpiLCs + PRDM14 treated with DMSO(DMSO; control), 3-aminobenzamide (3AB), or CRT 004876 (CRT). Error bars indicate ±SD of a technical duplicate.
(H) ChIP-qPCR analysis of PRDM14 (α- FLAG) and OCT3/4 at the *Klf2* locus in EpiLCs + PRDM14 treated with DMSO, 3AB, or CRT. Fold enrichment was calculated by comparing pre-immune IgG control (red dotted line) with each antibody. Error bars indicate +SD of a technical duplicate.

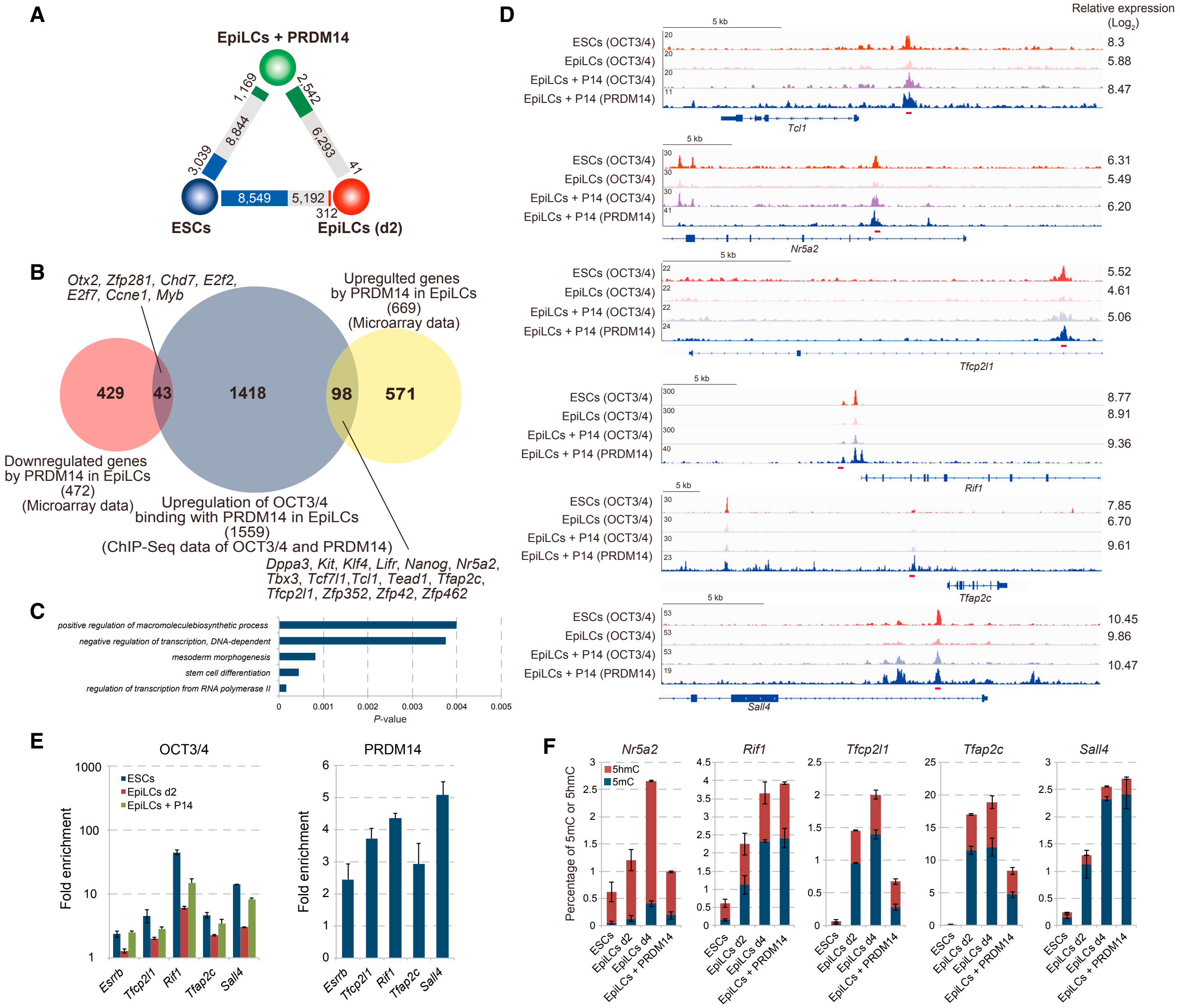

Figure 6. Enhancement of OCT3/4 Binding by PRDM14 during Reversion from Primed to Naive Pluripotency

(A) Comparison of OCT3/4 binding peaks between ESCs and EpiLCs (d2), EpiLCs (d2) and EpiLCs + PRDM14, and EpiLCs + PRDM14 and ESCs. Numbers in gray rectangles indicate peaks common to both cell types in each pair. Numbers in blue, red, and green rectangles indicate peaks specific to ESCs, EpiLCs (d2), and EpiLCs + PRDM14, respectively.

(B) Genes downregulated by PRDM14 during the transition from EpiLCs to ESCLCs (red circles). Genes associated with PRDM14 and OCT3/4 binding that were enhanced during the transition of EpiLCs to ESCs are indicated by navy circles; genes upregulated by PRDM14 in EpiLCs are shown by yellow circles.

(C) GO annotation of genes upregulated by PRDM14 in which OCT3/4 binding increased with PRDM14 binding (from EpiLCs to EpiLCs + PRDM14).

(D) ChIP-seq tracks of OCT3/4 and PRDM14 in ESCs, EpiLCs (d2), and EpiLCs + PRDM14. Intensity values of microarray data are shown on the right side. Red bar indicates the position of the primers for ChIP-qPCR and GlucMS-qPCR.

(E) ChIP-qPCR analysis of OCT3/4 and PRDM14 expression in ESCs, EpiLCs (d2), and EpiLCs + PRDM14. Fold enrichment indicates values of each IgG relative to input values using the same amount of DNA as template. Error bars indicate ±SD of a technical duplicate.

(F) GlucMS-qPCR analysis of the percentage of 5mC and 5hmC marks at pluripotency genes. Error bars indicate ±SD of a technical duplicate.

PRDM14 were downregulated by *Prdm14* induction in EpiLCs (Figure 6B). An overrepresentation analysis using DAVID (Huang et al., 2007) revealed that GO terms related to stem cell maintenance, including *Tcl1*, *Nr5a2*, and *Tfcp2l1*, were enriched among the upregulated genes (Figures 6C and 6D). Consistent with ChIP-seq data, the

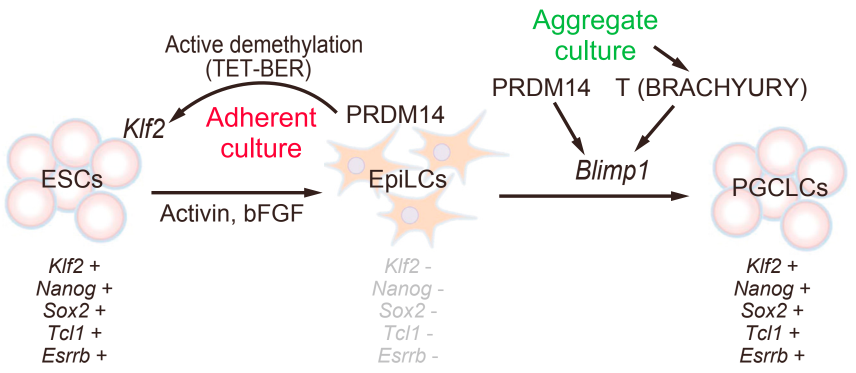

Figure 7. Working Model of the Role of PRDM14 in the Conversion of EpiLCs to ESCLCs and PGC Specification

enrichment of OCT3/4 binding at five of the genes decreased during differentiation of ESC to EpiLC, with a consequent increase in OCT3/4 binding peaks by PRDM14 binding-associated induction (Figure 6E). To determine whether PRDM14-mediated DNA demethylation modulates the recruitment of OCT3/4 to the five genes, we measured 5mC and 5hmC levels at the binding regions of OCT3/4 and PRDM14 by glucosylation of genomic DNA followed by methylation-sensitive (GlucMS)-qPCR (Figure 6F). 5mC levels were elevated at all genes analyzed during ESC-to-EpiLC differentiation, whereas at three of the five genes the levels decreased after PRDM14 induction in EpiLCs. These findings indicate that PRDM14 acts via DNA demethylation-dependent and -independent mechanisms to recruit OCT3/4.

DISCUSSION

This study provides evidence that PRDM14 confers pluripotency and self-renewal capacity to EpiLCs by reactivating pluripotency genes via TET-BER-mediated active demethylation (Figure 7). Nascent PGCs reactivate pluripotent-associated genes during specification from epiblast cells. Our data suggest that the activation of PRDM14 in epiblast is a key event for reactivation of pluripotent-associated genes and the acquisition of potential pluripotency. Our data demonstrate that EpiLC culture conditions (i.e., adherent versus aggregate) determine the expression of two key genes for PGC specification, *T* and *Blimp1* (Ohinata et al., 2005) (Figures 2G and 2H). Moreover, adherent EpiLC cultures with *Prdm14* induction resulted in the conversion of EpiLCs to ESCLCs, whereas three-dimensional aggregate cultures induced PGCLCs (Nakaki et al., 2013). It has been shown that the WNT3-β-catenin axis induces *T* expression in epiblast culture in aggregate culture of EpiLCs (Aramaki et al., 2013). Therefore, we consider that aggregate culture of EpiLCs enhance WNT3-β-catenin signaling to activate *T* expression. Further experiments are needed to clarify whether the combination of PRDM14 with BLIMP1 or T can induce PGCLCs from EpiLCs in adherent culture.

Tet1/*Tet2* KD and BERi treatment impaired the reactivation of pluripotency genes by PRDM14, reducing the conversion efficiency of EpiLCs to ESCLCs. However, the reactivation of pluripotency genes by PRDM14 was partially inhibited by *Tet1*/*Tet2* KD and BERi treatment, implying that PRDM14 acts via a TET-BER-independent mechanism in this process. Furthermore, PRDM14-induced activation of PGC-enriched genes including *Tfap2c*, developmental pluripotency-associated 3 (*Dppa3*), and reproductive homeobox 6/9 (*Rhox6/9*) was normal in *Tet1*/*Tet2* KD EpiLCs. These data are consistent with the phenotype of *Tet1*/*Tet2* double KO (DKO) PGCs, and demonstrate normal PGC specification and development (Yamaguchi et al., 2012). Further studies examining the developmental potential of EGCs derived from *Tet1*/*Tet2* DKO PGCs are needed to clarify the function of TET1/TET2 in the acquisition of pluripotency by PGCs in vivo.

The concordance between ChIP-seq and microarray data provided evidence that PRDM14 recruits OCT3/4 to enhancer regions of pluripotency genes, leading to their transcription during the conversion from EpiLCs to ESCLCs. The 5mC marks at *Nr5a2*, *Tfcp2l1*, and *Tfap2c* enhancers were removed by PRDM14 in EpiLCs, while 5mC at replication timing regulatory factor 1 (*Rif1*) and spalt-like transcription factor 4 (*Sall4*) enhancers were unchanged. Interestingly, the reactivation of *Rif1* and *Sall4* by PRDM14 was impaired in *Tet1*/*Tet2* double KD EpiLCs (Figure S4B). These results suggest that TET proteins are involved in the reactivation of *Rif1* and *Sall4* by PRDM14 via DNA demethylation-independent mechanisms. In support of this possibility, TET proteins were required for the recruitment of O-linked N-acetylglucosamine (O-GlcNAc) transferase to target chromatin, which leads to its activation via O-GlucNAcylation of histone H2B (Chen et al., 2013; Vella et al., 2013).

Recently, various groups have independently succeeded in inducing human PGCLCs from human pluripotent

stem cells (PSCs) (Irie et al., 2015; Sasaki et al., 2015; Sugawa et al., 2015). For instance, human PGCLCs were more robustly induced from mesoderm-like cells (iMeLCs) and human ESCs cultured in medium containing four inhibitors, called "4i" medium; they expressed some mesoderm markers. Although most naive pluripotency genes are repressed in the transition from ESCs to EpiLCs in mice, most genes including *KLF2*, *TCL1A*, *ESRRB*, *TFCP2L1*, and nuclear receptor subfamily 0 B1 (*NR0B1*) showed low expression in human ESCs/induced PSCs (iPSCs). Although expression of *Prdm14* is closely associated with naive pluripotency in mice, primed human ESCs/iPSCs, primitive streak-like cells, iMeLCs, and 4i ESCs consistently express PRDM14 at moderate levels. Therefore, the specific upregulation of *Prdm14* observed during the transition from pluripotent cells to PGCs in mice does not occur in human PGC specification. The present study has clearly demonstrated that PRDM14 governs the transcriptional network for pluripotency in the absence of BLIMP1 in mice. In contrast to early mouse embryos, epiblast cells in human early embryos need to maintain pluripotency for longer than 10 days until the emergence of PGCs. Furthermore, primitive streak-like cells, which have a high capacity for PGC specification, retain pluripotency markers including NANOG and PRDM14 (Irie et al., 2015; Sasaki et al., 2015). On this basis, we hypothesize that continuous expression of PRDM14 is essential for the long-term maintenance of pluripotent cells to give rise to PGCs in humans. In contrast to our hypothesis, it has also been shown that PRDM14 KD ESCs are competent for PGCLC induction, which led to the conclusion that PRDM14 is not necessary for PGC induction in humans (Sugawa et al., 2015). However, another group has clearly shown that *PRDM14* KD induces human ESC differentiation (Chia et al., 2010), implying that the KD efficiency in the study of Sugawa et al. (2015) was quite low and that conclusions cannot be drawn regarding the role of PRDM14 in human PGC development. Therefore, additional KO studies are needed to clarify the molecular and epigenetic functions of PRDM14 in the human germline.

The conversion of EpiLCs to ESCLCs is similar to the late stage of somatic cell reprogramming, since activation of pluripotency genes by PRDM14 is associated with DNA demethylation of their promoters. Given these findings, we consider that PRDM14-dependent active demethylation of pluripotency genes is required for progression of the late stage of somatic cell reprogramming. This is supported by the fact that *Prdm14* KO fibroblasts failed to be converted to iPSCs in the absence of *Nanog* and *Sox2* reactivation under standard ESC culture conditions (Payer et al., 2013). Furthermore, epigenetic errors result in partially reprogrammed iPSCs and low-grade iPSCs (Stadtfeld et al., 2010; Tran et al., 2015). Our findings provide a framework for understanding not only physiological but also artificial (e.g., iPSC derivation) and pathological (e.g., the appearance of cancer stem cells) reprogramming.

EXPERIMENTAL PROCEDURES

Cell Culture

E14tg2a ESCs were cultured in Glasgow minimum essential medium (Wako) containing 10% fetal calf serum (Invitrogen), 1 mM glutamine (Wako), nonessential amino acid (Wako), and 0.1 mM 2-mercaptoethanol (Wako). The culture was supplemented with LIF (Wako) in the absence of feeder cells.

Teratoma Formation and Histological Analysis

Approximately 5×10^6 EpiLCs expressing PRDM14, cultured without LIF, were injected subcutaneously into both the flanks of female nude mice. After 30 days, the tumors were excised, embedded in paraffin, and sectioned into 4-μm-thick slices. H&E staining was done according to the standard protocol. Markers for the three germ layers were monitored with immunofluorescence with antibodies: α-AFP (Proteintech; 14550-1-AP), α-SMA (Abcam; ab5694), and α-β-tubulin (Cell Signaling; catalog no. 2146). These experiments were conducted according to the Kwansei Gakuin University Regulation for Animal experimentation.

Generation of *Prdm14*-Inducible ESCs by ROSA-Tet-Off and piggyBac Tet-On

For the ROSA-Tet-off system, EBRTcH3 cells were transfected with pZhc-Prdm14 and pCAGGS-Cre (Masui et al., 2005). For the Tet-on system, E14tg2a ESCs and BVSC ESCs (Hayashi et al., 2011) were transfected with PB-TET-FLAG-*Prdm14*-IRES-Neo, PB-CA-rtTA Adv, pCAG-Pbase, and pGG131 (pCAG-DsRed-IRES-Hygro). The cells were grown in selective medium containing 1.5 μg/mL puromycin (Sigma-Aldrich) and 200 μg/mL hygromycin B (Wako) for 7 days (Okashita et al., 2014). Colonies were picked and analyzed by qRT-PCR and western blotting to determine PRDM14 expression levels, with and without doxycycline.

RNAi

Tet1 and *Tet2* small hairpin RNAs (shRNAs) were generated from pLKO.1-puro (Addgene; 8453) or pLKO.1-blast (Addgene; 26655). The *Tet1* and *Tet2* shRNAs were adapted for pLKO.1 plasmids. The RNAi target sequences are presented in Table S1. pLKO.1 lentiviruses were constructed according to the Addgene pLKO.1 protocol (http://www.addgene.org/). pLKO.1 plasmids were co-transfected with pCMV-VSV-G (Addgene; 8454) and pCMV-dR8.2 dvpr (Addgene; 8455) into HEK293T cells. Lentivirus-containing supernatants were centrifuge-filtered through a 0.45-μm filter. Clarified supernatants were combined using the Lenti-X Concentrator (Clontech) according to the manufacturer's protocol. The mixture was centrifuged, after which the supernatant was carefully removed and the pellet resuspended in DMEM.

For lentivirus transduction, subconfluent iP14 ESCs were incubated with lentivirus-containing DMEM supplemented with 8 μg/mL polybrene (Sigma-Aldrich) for 24 hr. After changing the spent medium with fresh medium, cells were selected with

2 μg/mL puromycin (Sigma-Aldrich) or 20 μg/mL blasticidin S hydrochloride (Calbiochem). Knockdown efficiency was analyzed by qRT-PCR and western blotting.

Generation of *Klf2*-Knockout ESCs Using a CRISPR/Cas9 System

Klf2 guide RNA was adapted for and generated from the pX330-U6-Chimeric_BB-CBh-hSpCas9 plasmid (Addgene; 42230). The *Klf2* guide RNA sequences are presented in Table S1. The established plasmid was co-transfected with pCAG-IRES-Puro into the induced PRDM14 ESCs, and the transfected cells were selected with 2 μg/mL puromycin. KLF2 expression was analyzed by western blot.

Induction of EpiLCs

ESCs were cultured in N2B27 basal medium containing 3 μM CHIR99021 (Wako), 0.4 μM PD0325901 (Wako), and LIF in a dish coated with 0.01% poly-L-ornithine (Millipore) and 10 ng/mL laminin (BD Biosciences). Induction of EpiLCs was performed as described previously (Hayashi et al., 2011). EpiLCs were induced from ESCs in N2B27 basal medium containing 20 ng/mL activin A (Peprotech), 12 ng/mL bFGF (Invitrogen), and 0.1–1% KSR for 2 days on a dish coated with 16.7 μg/mL human plasma fibronectin (Millipore). Two days after EpiLC induction, the cells were collected and replated (0.5–1.0 × 10^5) with or without doxycycline in GK15 medium on a dish coated with 16.7 μg/mL human plasma fibronectin. For the colony formation assay for AP-positive cells, ESCs, EpiLCs, and EpiLCs induced with or without PRDM14 were dissociated by TrypLE Select (Invitrogen). Cells (1.0 × 10^4) were cultured under standard ESC culture conditions. After culture for 3 days, the cells were stained for AP activity. The aggregate culture of EpiLCs was performed as previously reported (Nakaki et al., 2013). In brief, after 36 hr of EpiLC induction the cells were collected, and 2,000 cells per well were transferred to a low-cell binding 96-well plate (NUNC). The cells were grown for 2 days in GK15 medium with or without 1.5 μg/mL doxycycline.

qRT-PCR

Total RNA was extracted using TRIzol (Invitrogen). The ReverTra Ace qPCR RT kit (Toyobo) was used for cDNA synthesis according to the manufacturer's instructions. Subsequently, cDNA was used as a template for qPCR with the Thunderbird SYBR qPCR Mix (Toyobo) and gene-specific primers (Table S1) on a LightCycler 96 system (Roche).

Western Blot and Immunoprecipitation Analysis

Cells were lysed by boiling in SDS sample buffer (Wako). The total proteins from lysed cells were denatured by 2-mercaptoethanol and were then applied to polyacrylamide-SDS gels. The proteins were separated on polyacrylamide-SDS gels, blotted on polyvinylidene fluoride membrane, and probed using the following primary antibodies: α-PRDM14 (R & D Systems; MAB8097), α-tubulin (Sigma; T5168), α-histone H3 (Abcam; ab1791), α-FLAG (Sigma; F1804), α-KLF2 (Millipore; 09–820), α-TET1 (Millipore; 09–872), α-TET2 (Santa Cruz Biotechnology; sc-136926), and α-OCT3/4 (Santa Cruz; sc-8628). Following the primary antibody reaction, the membrane was incubated with secondary horseradish peroxidase-coupled antibodies. Detection was achieved using the Luminata Forte Western HRP Substrate (Millipore). For immunoprecipitation analysis, extracts containing a protease inhibitor cocktail (Roche) were incubated with anti-PRDM14 antibody or anti-OCT3/4 antibody for 2 hr at 4°C, and captured with protein A beads. Protein complexes were washed with wash buffer (50 mM Tris-HCl [pH 8.0], 150 mM NaCl, 1% NP-40), and eluted by boiling with SDS sample buffer.

ACCESSION NUMBERS

Data from this study are available in GEO: GSE77622 (Microarray) and in DNA DataBank of Japan (DDBJ): DRA004294 (ChIP-seq).

SUPPLEMENTAL INFORMATION

Supplemental Information includes Supplemental Experimental Procedures, five figures, and one table and can be found with this article online at http://dx.doi.org/10.1016/j.stemcr.2016.10.007.

AUTHOR CONTRIBUTIONS

N.O. performed most experiments and analyzed data. Y. Suwa, N.S., M.K., H.K., and A.N. performed some experiments. O.N. and M.K. contributed ChIP-seq analysis. G.N. established ESC cell line. Y. Seki designed and performed experiments. M.T. contributed to the immunofluorescence study of teratoma tissue. N.O. and Y. Seki wrote the manuscript. Y. Suwa, O.N., and N.S. contributed equally to this work.

ACKNOWLEDGMENTS

We thank all members of the Seki laboratory for stimulating discussions. We thank M. Saitou for providing the BVSC ES cell line. We thank C. Tanegashima and K. Tatsumi for technical assistance with the ChIP-seq experiment. We also thank S. Kuraku for his advice on the ChIP-seq experiment. Y. Seki thanks Andrew Johnson for stimulating discussions. This study was supported by a Grant-in-Aid for Young Scientists (A) (KAKENHI) (grant number: 24681040), Scientific Research on Innovative Areas, 'Epigenome dynamics and regulation in germ cells' (grant numbers 26112514, 16H01223), Scientific Research on Innovative Areas, 'Mechanisms regulating gamete formation in animals' (grant number 16H01258), the Takeda Science Foundation, and the Uehara Memorial Foundation.

Received: February 18, 2016
Revised: October 17, 2016
Accepted: October 18, 2016
Published: November 17, 2016

REFERENCES

Aramaki, S., Hayashi, K., Kurimoto, K., Ohta, H., Yabuta, Y., Iwanari, H., Mochizuki, Y., Hamakubo, T., Kato, Y., Shirahige, K., et al. (2013). A mesodermal factor, T, specifies mouse germ cell fate by directly activating germline determinants. Dev. Cell *27*, 516–529.

Buecker, C., Srinivasan, R., Wu, Z., Calo, E., Acampora, D., Faial, T., Simeone, A., Tan, M., Swigut, T., and Wysocka, J. (2014). Reorganization of enhancer patterns in transition from naive to primed pluripotency. Cell Stem Cell *14*, 838–853.

Chen, Q., Chen, Y., Bian, C., Fujiki, R., and Yu, X. (2013). TET2 promotes histone O-GlcNAcylation during gene transcription. Nature *493*, 561–564.

Chia, N.Y., Chan, Y.S., Feng, B., Lu, X., Orlov, Y.L., Moreau, D., Kumar, P., Yang, L., Jiang, J., Lau, M.S., et al. (2010). A genome-wide RNAi screen reveals determinants of human embryonic stem cell identity. Nature *468*, 316–320.

Feng, J., Liu, T., Qin, B., Zhang, Y., and Liu, X.S. (2012). Identifying ChIP-seq enrichment using MACS. Nat. Protoc. *7*, 1728–1740.

Ficz, G., Hore, T.A., Santos, F., Lee, H.J., Dean, W., Arand, J., Krueger, F., Oxley, D., Paul, Y.L., Walter, J., et al. (2013). FGF signaling inhibition in ESCs drives rapid genome-wide demethylation to the epigenetic ground state of pluripotency. Cell Stem Cell *13*, 351–359.

Gillich, A., Bao, S., Grabole, N., Hayashi, K., Trotter, M.W., Pasque, V., Magnusdottir, E., and Surani, M.A. (2012). Epiblast stem cell-based system reveals reprogramming synergy of germline factors. Cell Stem Cell *10*, 425–439.

Hackett, J.A., Zylicz, J.J., and Surani, M.A. (2012). Parallel mechanisms of epigenetic reprogramming in the germline. Trends Genetics *28*, 164–174.

Hajkova, P., Ancelin, K., Waldmann, T., Lacoste, N., Lange, U.C., Cesari, F., Lee, C., Almouzni, G., Schneider, R., and Surani, M.A. (2008). Chromatin dynamics during epigenetic reprogramming in the mouse germ line. Nature *452*, 877–881.

Hall, J., Guo, G., Wray, J., Eyres, I., Nichols, J., Grotewold, L., Morfopoulou, S., Humphreys, P., Mansfield, W., Walker, R., et al. (2009). Oct4 and LIF/Stat3 additively induce Kruppel factors to sustain embryonic stem cell self-renewal. Cell Stem Cell *5*, 597–609.

Hayashi, K., Ohta, H., Kurimoto, K., Aramaki, S., and Saitou, M. (2011). Reconstitution of the mouse germ cell specification pathway in culture by pluripotent stem cells. Cell *146*, 519–532.

Huang, D.W., Sherman, B.T., Tan, Q., Kir, J., Liu, D., Bryant, D., Guo, Y., Stephens, R., Baseler, M.W., Lane, H.C., et al. (2007). DAVID Bioinformatics Resources: expanded annotation database and novel algorithms to better extract biology from large gene lists. Nucleic Acids Res. *35*, W169–W175.

Irie, N., Weinberger, L., Tang, W.W., Kobayashi, T., Viukov, S., Manor, Y.S., Dietmann, S., Hanna, J.H., and Surani, M.A. (2015). SOX17 is a critical specifier of human primordial germ cell fate. Cell *160*, 253–268.

Kagiwada, S., Kurimoto, K., Hirota, T., Yamaji, M., and Saitou, M. (2013). Replication-coupled passive DNA demethylation for the erasure of genome imprints in mice. EMBO J. *32*, 340–353.

Kawasaki, Y., Lee, J., Matsuzawa, A., Kohda, T., Kaneko-Ishino, T., and Ishino, F. (2014). Active DNA demethylation is required for complete imprint erasure in primordial germ cells. Sci. Rep. *4*, 3658.

Kurimoto, K., Yabuta, Y., Ohinata, Y., Shigeta, M., Yamanaka, K., and Saitou, M. (2008). Complex genome-wide transcription dynamics orchestrated by Blimp1 for the specification of the germ cell lineage in mice. Genes Dev. *22*, 1617–1635.

Kurimoto, K., Yabuta, Y., Hayashi, K., Ohta, H., Kiyonari, H., Mitani, T., Moritoki, Y., Kohri, K., Kimura, H., Yamamoto, T., et al. (2015). Quantitative dynamics of chromatin remodeling during germ cell specification from mouse embryonic stem cells. Cell Stem Cell *16*, 517–532.

Ma, Z., Swigut, T., Valouev, A., Rada-Iglesias, A., and Wysocka, J. (2011). Sequence-specific regulator Prdm14 safeguards mouse ESCs from entering extraembryonic endoderm fates. Nat. Struct. Mol. Biol. *18*, 120–127.

Masui, S., Shimosato, D., Toyooka, Y., Yagi, R., Takahashi, K., and Niwa, H. (2005). An efficient system to establish multiple embryonic stem cell lines carrying an inducible expression unit. Nucleic Acids Res. *33*, e43.

Matsui, Y., Zsebo, K., and Hogan, B.L. (1992). Derivation of pluripotential embryonic stem cells from murine primordial germ cells in culture. Cell *70*, 841–847.

Nakaki, F., Hayashi, K., Ohta, H., Kurimoto, K., Yabuta, Y., and Saitou, M. (2013). Induction of mouse germ-cell fate by transcription factors in vitro. Nature *501*, 222–226.

Ohinata, Y., Payer, B., O'Carroll, D., Ancelin, K., Ono, Y., Sano, M., Barton, S.C., Obukhanych, T., Nussenzweig, M., Tarakhovsky, A., et al. (2005). Blimp1 is a critical determinant of the germ cell lineage in mice. Nature *436*, 207–213.

Ohinata, Y., Ohta, H., Shigeta, M., Yamanaka, K., Wakayama, T., and Saitou, M. (2009). A signaling principle for the specification of the germ cell lineage in mice. Cell *137*, 571–584.

Ohno, R., Nakayama, M., Naruse, C., Okashita, N., Takano, O., Tachibana, M., Asano, M., Saitou, M., and Seki, Y. (2013). A replication-dependent passive mechanism modulates DNA demethylation in mouse primordial germ cells. Development *140*, 2892–2903.

Okashita, N., Kumaki, Y., Ebi, K., Nishi, M., Okamoto, Y., Nakayama, M., Hashimoto, S., Nakamura, T., Sugasawa, K., Kojima, N., et al. (2014). PRDM14 promotes active DNA demethylation through the ten-eleven translocation (TET)-mediated base excision repair pathway in embryonic stem cells. Development *141*, 269–280.

Payer, B., Rosenberg, M., Yamaji, M., Yabuta, Y., Koyanagi-Aoi, M., Hayashi, K., Yamanaka, S., Saitou, M., and Lee, J.T. (2013). Tsix RNA and the germline factor, PRDM14, link X reactivation and stem cell reprogramming. Mol. Cell *52*, 805–818.

Popp, C., Dean, W., Feng, S., Cokus, S.J., Andrews, S., Pellegrini, M., Jacobsen, S.E., and Reik, W. (2010). Genome-wide erasure of DNA methylation in mouse primordial germ cells is affected by AID deficiency. Nature *463*, 1101–1105.

Respuela, P., Nikolic, M., Tan, M., Frommolt, P., Zhao, Y., Wysocka, J., and Rada-Iglesias, A. (2016). Foxd3 promotes exit from naive pluripotency through enhancer decommissioning and inhibits germline specification. Cell Stem Cell *18*, 118–133.

Sasaki, K., Yokobayashi, S., Nakamura, T., Okamoto, I., Yabuta, Y., Kurimoto, K., Ohta, H., Moritoki, Y., Iwatani, C., Tsuchiya, H., et al. (2015). Robust in vitro induction of human germ cell fate from pluripotent stem cells. Cell Stem Cell *17*, 178–194.

Seisenberger, S., Andrews, S., Krueger, F., Arand, J., Walter, J., Santos, F., Popp, C., Thienpont, B., Dean, W., and Reik, W. (2012). The dynamics of genome-wide DNA methylation reprogramming in mouse primordial germ cells. Mol. Cell *48*, 849–862.

Seki, Y., Hayashi, K., Itoh, K., Mizugaki, M., Saitou, M., and Matsui, Y. (2005). Extensive and orderly reprogramming of genome-wide chromatin modifications associated with specification and early development of germ cells in mice. Dev. Biol. *278*, 440–458.

Seki, Y., Yamaji, M., Yabuta, Y., Sano, M., Shigeta, M., Matsui, Y., Saga, Y., Tachibana, M., Shinkai, Y., and Saitou, M. (2007). Cellular dynamics associated with the genome-wide epigenetic reprogramming in migrating primordial germ cells in mice. Development *134*, 2627–2638.

Stadtfeld, M., Apostolou, E., Akutsu, H., Fukuda, A., Follett, P., Natesan, S., Kono, T., Shioda, T., and Hochedlinger, K. (2010). Aberrant silencing of imprinted genes on chromosome 12qF1 in mouse induced pluripotent stem cells. Nature *465*, 175–181.

Sugawa, F., Arauzo-Bravo, M.J., Yoon, J., Kim, K.P., Aramaki, S., Wu, G., Stehling, M., Psathaki, O.E., Hubner, K., and Scholer, H.R. (2015). Human primordial germ cell commitment in vitro associates with a unique PRDM14 expression profile. EMBO J. *34*, 1009–1024.

Tran, K.A., Jackson, S.A., Olufs, Z.P., Zaidan, N.Z., Leng, N., Kendziorski, C., Roy, S., and Sridharan, R. (2015). Collaborative rewiring of the pluripotency network by chromatin and signalling modulating pathways. Nat. Commun. *6*, 6188.

Vella, P., Scelfo, A., Jammula, S., Chiacchiera, F., Williams, K., Cuomo, A., Roberto, A., Christensen, J., Bonaldi, T., Helin, K., et al. (2013). Tet proteins connect the O-linked N-acetylglucosamine transferase Ogt to chromatin in embryonic stem cells. Mol. Cell *49*, 645–656.

Weber, S., Eckert, D., Nettersheim, D., Gillis, A.J., Schafer, S., Kuckenberg, P., Ehlermann, J., Werling, U., Biermann, K., Looijenga, L.H., et al. (2010). Critical function of AP-2 gamma/TCFAP2C in mouse embryonic germ cell maintenance. Biol. Reprod. *82*, 214–223.

Wu, S.C., and Zhang, Y. (2010). Active DNA demethylation: many roads lead to Rome. Nat. Rev. Mol. Cell Biol. *11*, 607–620.

Yabuta, Y., Kurimoto, K., Ohinata, Y., Seki, Y., and Saitou, M. (2006). Gene expression dynamics during germline specification in mice identified by quantitative single-cell gene expression profiling. Biol. Reprod. *75*, 705–716.

Yamaguchi, S., Hong, K., Liu, R., Shen, L., Inoue, A., Diep, D., Zhang, K., and Zhang, Y. (2012). Tet1 controls meiosis by regulating meiotic gene expression. Nature *492*, 443–447.

Yamaji, M., Seki, Y., Kurimoto, K., Yabuta, Y., Yuasa, M., Shigeta, M., Yamanaka, K., Ohinata, Y., and Saitou, M. (2008). Critical function of Prdm14 for the establishment of the germ cell lineage in mice. Nat. Genet. *40*, 1016–1022.

Stem Cell Reports, Volume 7

Supplemental Information

PRDM14 Drives OCT3/4 Recruitment via Active Demethylation in the Transition from Primed to Naive Pluripotency

Naoki Okashita, Yoshiaki Suwa, Osamu Nishimura, Nao Sakashita, Mitsutaka Kadota, Go Nagamatsu, Masanori Kawaguchi, Hiroki Kashida, Ayaka Nakajima, Makoto Tachibana, and Yoshiyuki Seki

Supplementary Figure S1

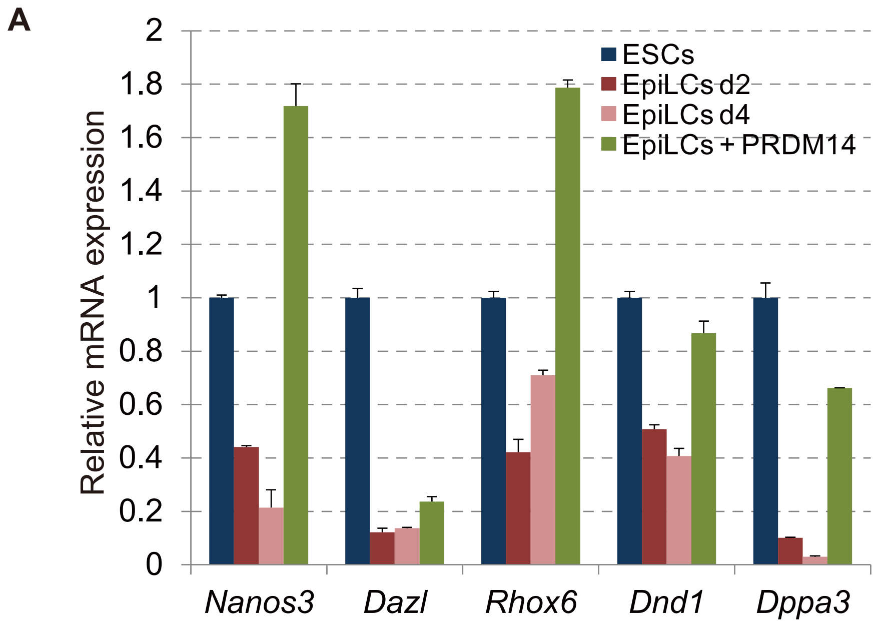

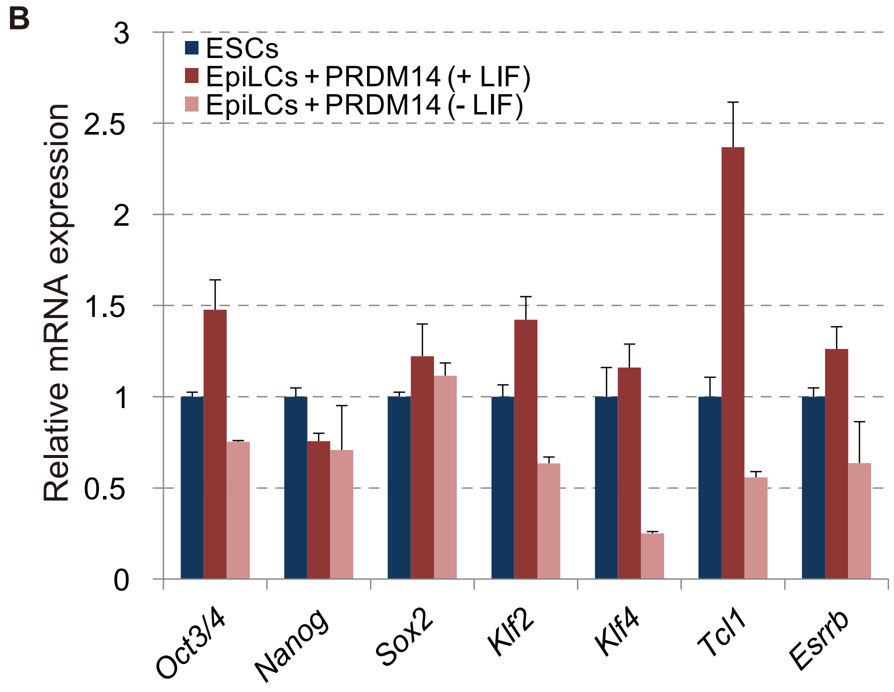

Supplementary Fig. 1 PRDM14 overexpression converts EpiLCs to ESC-like cells

(**A**) qRT-PCR anaysis of ealry PGC markers in ESCs, EpiLCs (d2), EpiLCs (d4) and EpiLCs + PRDM14. Error bar indicates + s.d. of technical duplicate. (**B**) qRT-PCR analysis of pluripotent-associated genes (*Oct3/4*, *Nanog*, *Sox2*, *Klf2*, *Klf4*, *Tcl1* and *Esrrb*) in ESCs and ESC-like cells converted from EpiLCs by PRDM14 in the presence or absence of LIF. Error bar indicates + s.d. of technical duplicate.

Supplementary Figure S2

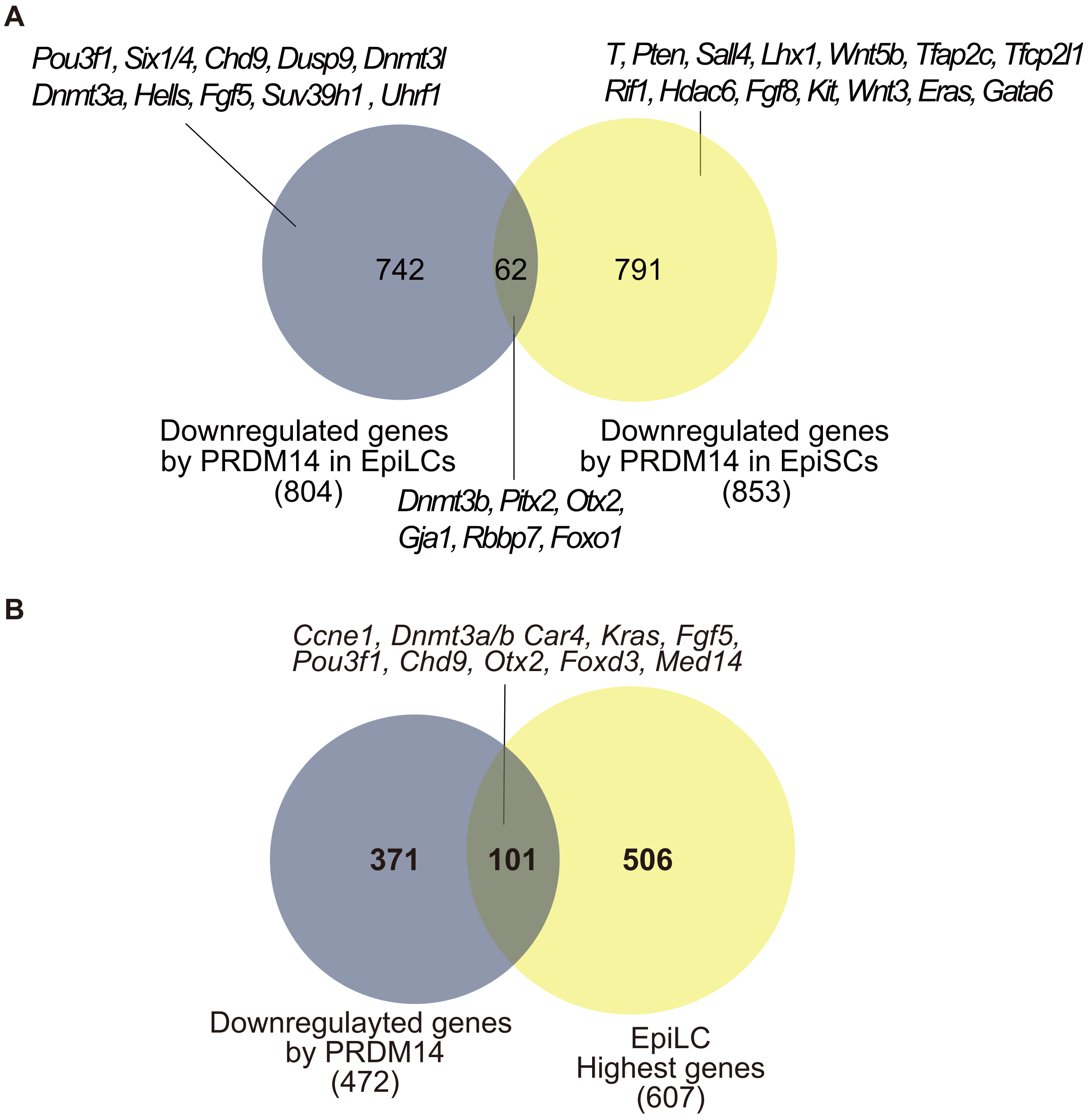

Supplementary Fig. 2 Venn diagram of downregulated genes by PRDM14

Supplementary Figure S3

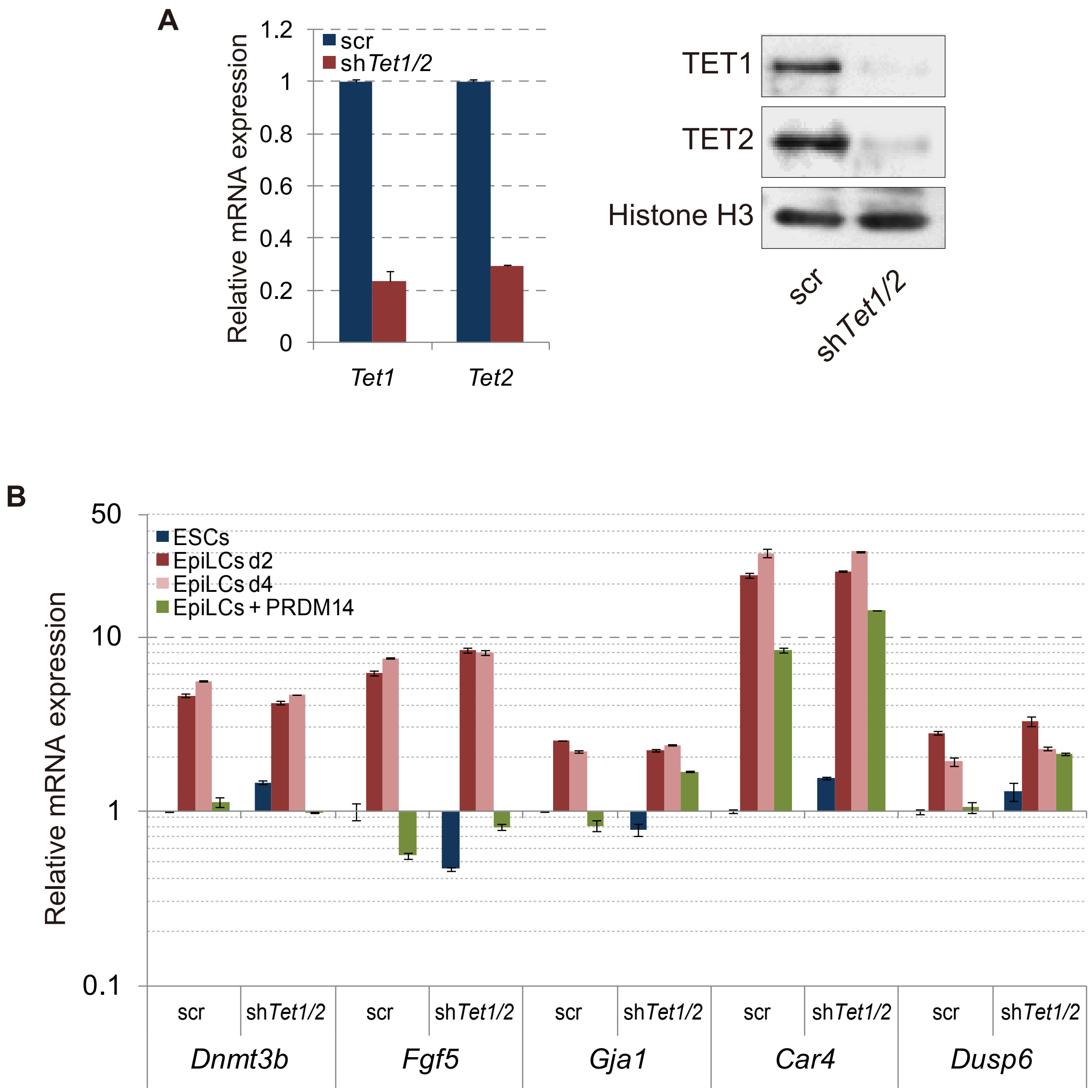

Supplementary Fig. 3 The varidation of knock down efficiency of *Tet1*, *Tet2* and qRT-PCR analysis

(**A**) qRT-PCR and Western blotting analysis of TET1 and TET2 expression in control (scr) and *Tet1/2* DKD (sh*Tet1/2*) Prdm14-inducible ESCs. (**B**) RT-PCR analysis of differentiation-associated genes and germline-specific genes expression in ESCs, EpiLCs d2, EpiLC d4 and EpiLCs + PRDM14 of control and *Tet1/Tet2* DKD cells. Error bar indicates ± s.d. of technical duplicate.

Supplementary Figure S4

A

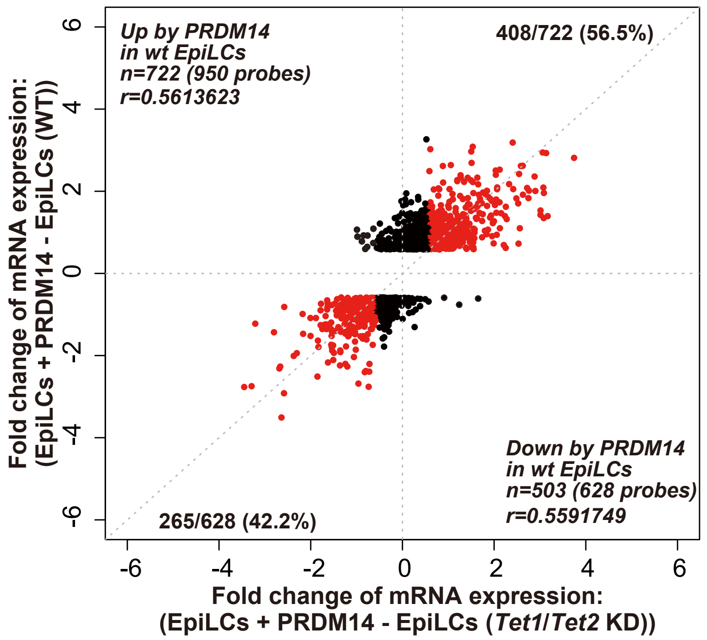

B

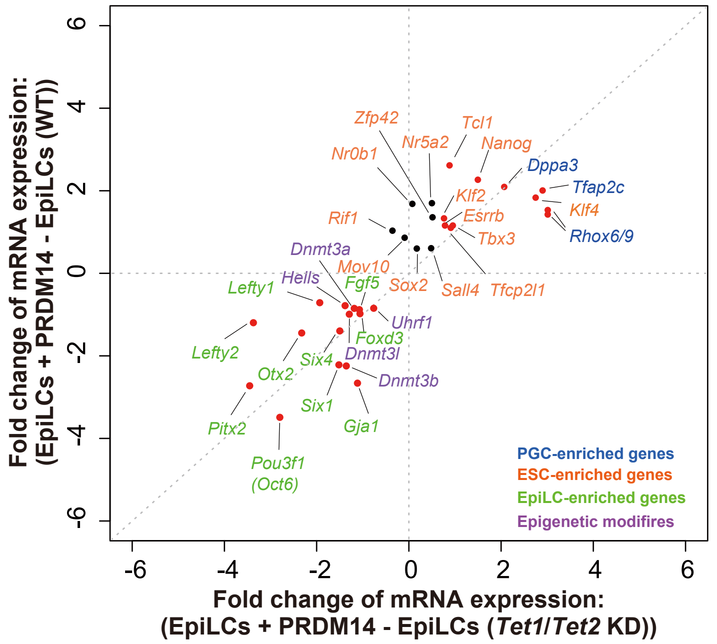

Supplementary Fig. 4 The effect of *Tet1*/*Tet2* KD in the conversion from EpiLCs to ESCLCs by PRDM14

(**A**, **B**) Scatterplot of microarray data represents genes upregulated or downregulated by PRDM14 in control and *Tet1*/*Tet2* KD EpiLCs. The y-axis indicates fold changes between + Dox control EpiLCs and –Dox control EpiLCs by applying ≥1.5-fold difference as a cut-off value. The x-axis indicates a fold change between + Dox *Tet1*/*Tet2* KD EpiLCs and –Dox *Tet1*/*Tet2* KD EpiLCs. Red dots indicate genes upregulated or downregulated by PRDM14 both in the control and *Tet1*/*Tet2* KD EpiLCs.

Supplementary Figure S5

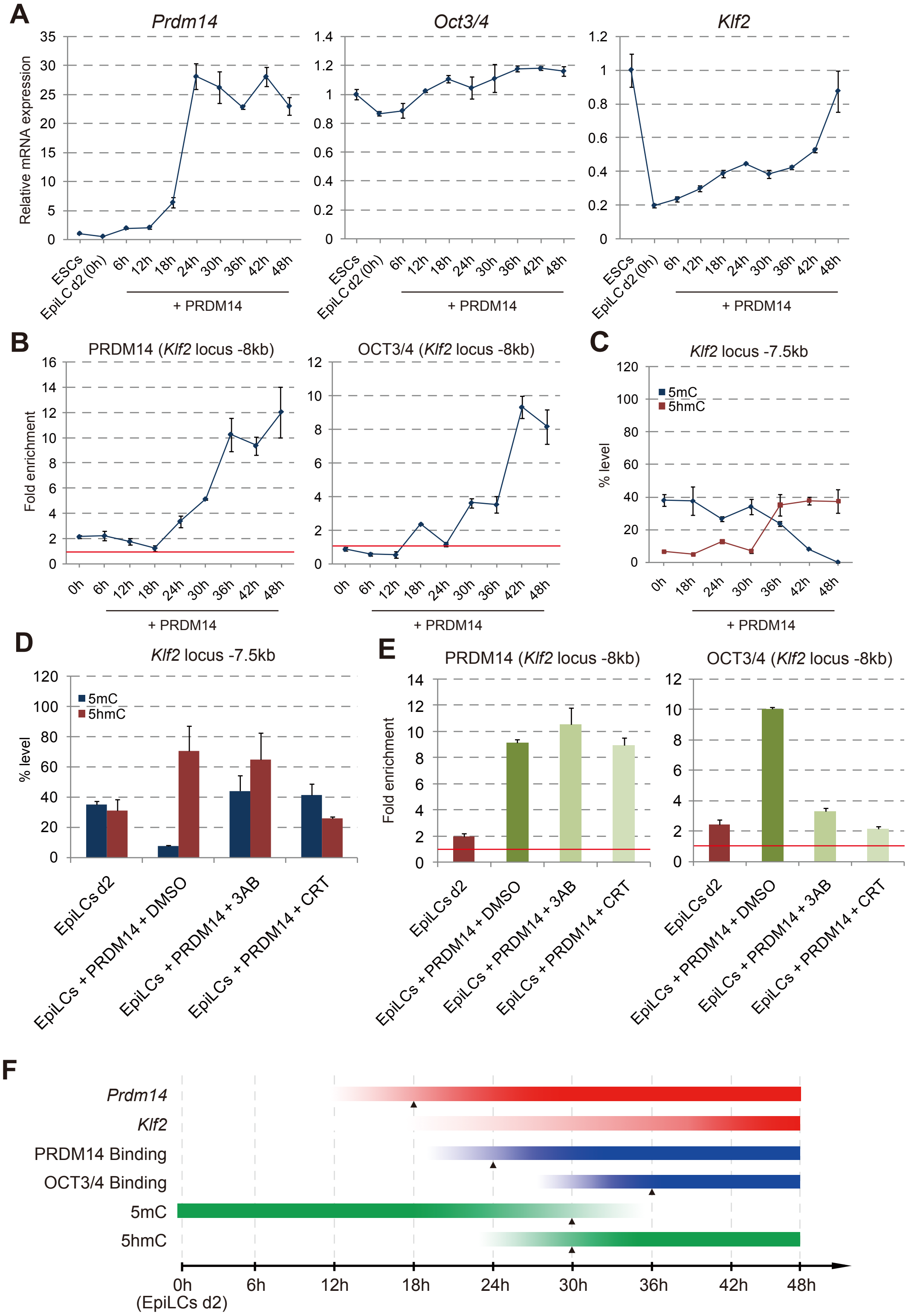

Supplementary Fig. 5 Molucular mechanism of *Klf2* activation by PRDM14

(**A**) qRT-PCR analysis of *Prdm14*, *Oct3/4* and *Klf2* in the conversion from EpiLCs to ESC-like cells by PRDM14. (**B**) ChIP analysis of PRDM14 and OCT3/4 on *Klf2* distal enhancer region in the conversion from EpiLCs to ESC-like cells by PRDM14. Fold enrichment values were calculated by comparing IgG control with PRDM14 and OCT3/4 antibody. (**C**) GlucMS-qPCR analysis of 5mC and 5hmC at *Klf2* distal enhancer region in the conversion from EpiLCs to ESC-like cells by PRDM14. (**D**) GlucMS-qPCR analysis of 5mC and 5hmC at neighboring *Klf2* distal enhancer region in EpiLCs d2 and EpiLCs d4 + PRDM14 (DMSO, 3AB, CRT). (**E**) ChIP analysis of PRDM14 and OCT3/4 on *Klf2* distal enhancer region in EpiLCs d2 and EpiLCs + PRDM14 (DMSO, 3AB, CRT). Fold enrichment values were calculated by comparing IgG control with PRDM14 and OCT3/4 antibody. Error bar indicates ± s.d. of technical duplicate of qPCR (**F**) A summary for the temporal hierarchy of events (PRDM14 and OCT3/4 binding, 5mC removal, and activation of *Klf2* expression)

Supplemental Experimental Procedures

Teratoma formation and histological analysis

Approximately 5 × 10^6 EpiLCs expressing PRDM14, cultured without LIF, were injected subcutaneously into both the flanks of female nude mice. After 30 days, the tumors were excised, embedded in paraffin, and sectioned into 4-μm-thick slices. Hematoxylin & Eosin staining was done according to the standard protocol. Markers for the three germ layers were monitored with immunofluorescence with antibodies: α-AFP (Proteintech Group, Inc.; 14550-1-AP), α-SMA (Abcam; ab5694), and α-β-Tubulin (Cell Signaling; #2146).

ChIP assay

Cells were cross-linked by adding 1% formaldehyde. The ChIP assay was carried out using the ChIP Assay kit (Upstate Biotechnology) according to the manufacturer's protocol (http://www.millipore.com/). Cross-linked protein/DNA was incubated with antibodies [α-FLAG (Sigma; F1804), α-OCT3/4 (N-19) (Santa Cruz; sc-8629)] for 12 hours at 4 °C with rotation. Antibody/protein/DNA complexes were collected by using

Dynabeads Protein A (Invitrogen) or Dynabeads Protein G (Invitrogen). De-fixed DNA was purified by phenol/chloroform extraction, followed by ethanol precipitation, and analyzed by qPCR. The primers used are presented in **Table S1**.

Expression microarray and data analysis

Total RNA was purified using the PureLink^{TM} RNA Mini Kit (Ambion). Purified total RNA (150 ng) was labeled using a GeneChip 3' IVT Express Kit (Affymetrix) and hybridized to MG-430 PM array strip performed on the GeneAtlas system (Affymetrix), according to the manufacturer's instructions. CELL files of GenChIP data were normalized by the Robust Multi-array Average (RMA) with the default setting in Affymetrix Expression Console^{TM}. Unsupervised hierarchical clustering (UHC) with Pearson correlation coefficient and scatter plot analysis was performed by using R program. Upregulated and downregulated genes by PRDM14 in EpiLCs was clustered using average linkage hierarchical clustering with Pearson correlation distance measure and visualized in a heatmap by Gene Cluster 3.0 and Java Treeview. Gene Ontology analysis was performed using DAVID version 6.7 (http://david.abcc.ncifcrf.gov/) with

default parameters. Venn diagrams were constructed using BioVenn(Hulsen et al., 2008).

Glucosylation of genomic DNA followed by methylation-sensitive (GlucMS)-qPCR analysis

Genomic DNA (2350 ng) was treated with T4 phage β-glucosyltransferase (T4-BGT) (NEB M0357S), according to the manufacturer's instructions. Glycosylated genomic DNA (750 ng) was digested with 40 U of either Hpa II, Msp I, or no enzyme at 37 °C overnight, followed by inactivation with proteinase K treatment. The Hpa II- or Msp I-resistant fractions were quantified by qPCR using primers designed around one Hpa II/Msp I site, and normalizing to the region lacking Hpa II/Msp I sites. Resistance to Msp I directly translated to percentage of 5hmC, whereas the percentage of 5mC was calculated by subtracting the 5hmC contribution from the total Hpa II resistance. The primers used are presented in **Table S1**.

Inhibition of the base excision repair pathway

Concurrently with the induction of PRDM14, small molecule inhibitors, either 5 mM 3-AB (Sigma-Aldrich) or 100 μM CRT0044876 (Calbiochem) of 0.1% DMSO, were added to the medium in order to inhibit the base excision repair pathway.

ChIP-seq and downstream *in silico* analysis

Library for the Illumina sequencing platform was prepared from 20 ng input DNA or 1 ng ChIP DNAs using the KAPA LTP library preparation kit (Kapa Biosystems) with Illumina TruSeq-compatible multiplex adapters. Quality control check for length distribution and concentration of DNA fragments in the prepared libraries was performed using the Agilent 2100 Bioanalyzer (Agilent Technologies). The prepared ChIP DNA libraries were sequenced in Rapid Run mode of HiSeq 1500 (Illumina) for 80 nt single-end reads, with 5% PhiX spike-in.

Raw sequence data were analyzed using FastQC program ver. 0.11.1 (http://www.bioinformatics.bbsrc.ac.uk/projects/fastqc/). Trimming of adapter sequences and the removal of low-quality reads was performed with the Trim Galore ver. 0.3.3 (http://www.bioinformatics.babraham.ac.uk/projects/trim_galore/) with

parameters ‘-e 0.1 -q 20’. The processed reads were aligned with the mouse genome sequences mm10 by Bowtie2(Langmead and Salzberg, 2012) ver. 2.2.5 with default parameters. Peak calling for individual ChIP-DNA libraries were performed by MACS2 ver. 2.1.0.20150316(Feng et al., 2012) with the filtering parameter as ‘-q 0.01’, using reads from input DNA library as control. To identify differentially enriched regions between two ChIP DNA libraries, the bdgdiff function of MACS2 was used. Evaluation and visualization of ChIP peaks was done by employing the UCSC Integrative Genomics Viewer (IGV) ver. 2.3.36(Thorvaldsdottir et al., 2013).

REFERENCES

Feng, J., Liu, T., Qin, B., Zhang, Y., and Liu, X.S. (2012). Identifying ChIP-seq enrichment using MACS. Nat Protoc *7*, 1728-1740.

Hulsen, T., de Vlieg, J., and Alkema, W. (2008). BioVenn - a web application for the comparison and visualization of biological lists using area-proportional Venn diagrams. BMC Genomics *9*, 488.

Langmead, B., and Salzberg, S.L. (2012). Fast gapped-read alignment with Bowtie 2. Nat Methods *9*, 357-359.

Thorvaldsdottir, H., Robinson, J.T., and Mesirov, J.P. (2013). Integrative Genomics Viewer (IGV): high-performance genomics data visualization and exploration. Brief Bioinform *14*, 178-192.
